# Cytoplasmic Ca^2+^ influx mediates iron- and reactive oxygen species-dependent ferroptotic cell death in rice immunity

**DOI:** 10.3389/fpls.2024.1339559

**Published:** 2024-05-02

**Authors:** Juan Wang, Won-Gyu Choi, Nam Khoa Nguyen, Dongping Liu, Su-Hwa Kim, Dongyeol Lim, Byung Kook Hwang, Nam-Soo Jwa

**Affiliations:** ^1^ Division of Integrative Bioscience and Biotechnology, College of Life Sciences, Sejong University, Seoul, Republic of Korea; ^2^ Department of Biochemistry and Molecular Biology, University of Nevada, Reno, NV, United States; ^3^ Department of Chemistry, College of Natural Sciences, Sejong University, Seoul, Republic of Korea; ^4^ Division of Biotechnology, College of Life Sciences and Biotechnology, Korea University, Seoul, Republic of Korea

**Keywords:** iron, ROS, Ca^2+^ influx, *GR* expression, ferroptotic cell death, rice, *Magnaporthe oryzae*

## Abstract

Iron- and reactive oxygen species (ROS)-dependent ferroptosis occurs in plant cells. Ca^2+^ acts as a conserved key mediator to control plant immune responses. Here, we report a novel role of cytoplasmic Ca^2+^ influx regulating ferroptotic cell death in rice immunity using pharmacological approaches. High Ca^2+^ influx triggered iron-dependent ROS accumulation, lipid peroxidation, and subsequent hypersensitive response (HR) cell death in rice (*Oryza sativa*). During *Magnaporthe oryzae* infection, 14 different Ca^2+^ influx regulators altered Ca^2+^, ROS and Fe^2+^ accumulation, *glutathione reductase* (*GR*) expression, glutathione (GSH) depletion and lipid peroxidation, leading to ferroptotic cell death in rice. High Ca^2+^ levels inhibited the reduction of glutathione isulphide (GSSG) to GSH *in vitro*. Ca^2+^ chelation by ethylene glycol-bis (2-aminoethylether)-*N, N, N’, N’*-tetra-acetic acid (EGTA) suppressed apoplastic Ca^2+^ influx in rice leaf sheaths during infection. Blocking apoplastic Ca^2+^ influx into the cytoplasm by Ca^2+^ chelation effectively suppressed Ca^2+^-mediated iron-dependent ROS accumulation and ferroptotic cell death. By contrast, acibenzolar-*S*-methyl (ASM), a plant defense activator, significantly enhanced Ca^2+^ influx, as well as ROS and iron accumulation to trigger ferroptotic cell death in rice. The cytoplasmic Ca^2+^ influx through calcium-permeable cation channels, including the putative resistosomes, could mediate iron- and ROS-dependent ferroptotic cell death under reduced *GR* expression levels in rice immune responses.

## Introduction

Plant cell death is an effective immune response to defend against microbial pathogens (Heath, 2000; Greenberg and Yao, 2004). Plant-pathogen interactions induce both pathogen-associated molecular patterns (PAMP)-triggered immunity (PTI) and effector-triggered immunity (ETI) in plant cells, depending on the mode of pathogen recognition (Jones and Dangl, 2006; Dangl et al., 2013; Ngou et al., 2021). Reactive oxygen species (ROS), such as superoxide, H_2_O_2_, and hydroxyl radical (•OH) are required to signal and execute plant cell death (Levine et al., 1994; Van Breusegem and Dat, 2006). ROS act as cellular signaling molecules that trigger PTI and ETI in plant cells after plants recognize pathogen infection (Jwa and Hwang, 2017). Robust ROS bursts are common signaling events that occur in hypersensitive response (HR) cell death (Van Breusegem and Dat, 2006; Jwa and Hwang, 2017). Virulent plant pathogens induce transient PTI with low levels of ROS. However, avirulent pathogens induce ETI with strong ROS bursts, leading to HR cell death (Grant and Loake, 2000). ETI is more potent in plant immunity than PTI and greatly limits the entry of microbial pathogens into plant cells via HR induced by intracellular nucleotide-binding leucine-rich repeat (NLR) receptors which can recognize pathogen effectors (Jones and Dangl, 2006). Plant pathogens have evolved to acquire a variety of effectors to suppress ROS bursts, which are key components of the plant immune response (Jwa and Hwang, 2017).

Ferroptosis is a nonapoptotic form of iron-dependent cell death first discovered in animal cells (Dixon et al., 2012; Stockwell et al., 2017) and then in plants, fungi, and bacteria (Distéfano et al., 2017; Dangol et al., 2019; Shen et al., 2020; Aguilera et al., 2022). Iron, ROS, and lipid hydroperoxides are directly involved in ferroptotic cell death (Stockwell et al., 2017; Dangol et al., 2019). The Fenton reaction (Fenton, 1894; Pierre and Fontecave, 1999) by iron ions (Fe^2+^) and ROS (H_2_O_2_) induces glutathione (GSH) depletion and iron- and ROS-dependent ferroptosis in the rice immune response (Dangol et al., 2019). Ca^2+^ is a conserved second messenger and a major mediator in plant immune responses (Köster et al., 2022). However, whether an abnormally high concentration of Ca^2+^ influx is directly associated with HR cell death is not fully understood, except for its role as a signal transducer in plant immunity (Moeder et al., 2019). Moreover, the role of Ca^2+^ in ferroptosis remains unclear.

Recently, it has been proposed that plant nucleotide-binding leucine-rich repeat receptors (NLRs) (Jones and Dangl, 2006; Dangl et al., 2013; Ngou et al., 2021) recognize pathogen effectors to form resistosome complexes as calcium-permeable cation channels in the plasma membrane (Bi et al., 2021). The ZAR1 (HOPZ-ACTIVATED RESISTANCE 1) resistosome (Lewis et al., 2020) is a membrane-localized Ca^2+^-permeable channel which can trigger immune signaling and cell death in *Arabidopsis* (Bi et al., 2021). The monocot wheat protein Sr35, which belongs to the CC-NLR class, has been demonstrated to assemble into a resistosome with a structure similar to ZAR1 (Förderer et al., 2022). *Arabidopsis* ‘helper’ immune NLRs form Ca^2+^-permeable cation channels, leading to cytoplasmic Ca^2+^ influx and subsequent cell death (Jacob et al., 2021). Ca^2+^-permeable cation channels of NLR-mediated resistosomes may induce a sustained high cytoplasmic Ca^2+^ influx during plant ETI (Jacob et al., 2021). Thus, the discovery of resistosomes that exhibit Ca^2+^ channel activity in plants provided a crucial clue to elucidate the common mechanism of plant cell death and immunity (Bi et al., 2021; Jacob et al., 2021).

The Ca^2+^ concentration in the apoplast (~1 mM) is approximately 10,000-fold higher than that in the cytoplasm (~100 nM) (Stael et al., 2012). The significant Ca^2+^ concentration gradient in the cell may be maintained by both passive impermeability of the plasma membrane to calcium ions and their active transport from the cytoplasm to the apoplast. Schanne et al. (1979) first demonstrated that perturbation of calcium homeostasis triggers toxic cell death in rat hepatocytes through cytoplasmic Ca^2+^ influx due to impaired membrane integrity, highlighting the pivotal role of Ca^2+^ in toxic cell death. During toxic cell death, disruption of Ca^2+^ homeostasis coincides with a decrease in glutathione peroxidase (GPX) and glutathione reductase (GR) activity, resulting in cellular damage due to the acute oxidative stress by glutathione depletion (Bellomo et al., 1982; Jewell et al., 1982). The other alternative hypothesis was that the cell death is not only due to ROS themselves, but also due to the generation of the hydroxyl radical, a more potent oxidizing species, through its reaction with iron (Starke and Farber, 1985; Farber, 1994). Reduced glutathione (GSH) plays a crucial role in controlling ROS in cells. ROS function as intracellular and extracellular signaling molecules. Complex crosstalks between ROS, oxidized glutathione (GSSG) and reduced glutathione (GSH), and the antioxidant enzyme glutathione reductase (GR) control the redox state inside the cell to be suitable for activation of programmed cell death (Couto et al., 2016). However, the roles of GPX and GR in plant HR cell death are largely unknown.

Rice (*Oryza sativa*)-*Magnaporthe oryzae* interaction is a good experimental system to analyze if Ca^2+^ mediates iron- and ROS-dependent ferroptotic cell death in plant immunity (Valent, 2021). In this study, Ca^2+^ sensor (*35S::GCaMP6mC*) transgenic rice lines, the Ca^2+^ indicator Fluo-5F AM, and the *o*-cresolphthalein complexone (*o*-CPC) method were used to visualize and quantify intracellular and apoplastic Ca^2+^ levels in rice leaf sheaths treated with multiple Ca^2+^ influx enhancers and/or inhibitors during *M. oryzae* infection. Taken together, our results suggest that Ca^2+^ influx from apoplast via calcium-permeable cation channels, triggers iron-dependent lipid-based reactive oxygen species (lipid ROS) accumulation by reduced *Glutathione Reductase* (*GR*) expression and glutathione depletion in rice cells, which acts as a critical redox switch for iron- and lipid ROS-dependent ferroptotic cell death.

## Materials and methods

### Plant material and growth conditions

Rice (*Oryza sativa L.*) cultivar Kitaake was used as the wild type (WT) in this study. Seeds of Kitaake were obtained from the National Institute of Crop Science, Jeonju, Korea (http://www.nics.go.kr). Plants were raised in a growth chamber at 30°C under 60% humidity and 16 h light/8 h dark photoperiod.

### *Magnaporthe oryzae* strains and culture conditions

*M. oryzae* strains RO1-1 and 007 were obtained from the Center for Fungal Genetic Resources, Seoul National University, Korea (http://genebank.snu.ac.kr). *M. oryzae* RO1-1 is virulent to rice cultivar Kitaake, whereas *M. oryzae* 007 is avirulent. Both *M. oryzae* strains were stored at −20°C and cultured on rice bran agar medium (20 g of rice bran, 20 g of sucrose, and 20 g of agar in 1 L of water) at 25°C in the dark for 10–14 days (Singh et al., 2016). Sporulation of *M. oryzae* cultures was induced by incubating the culture plates under continuous light for 3–4 days. *M. oryzae* conidia were harvested from the sporulated culture plates using 0.025% Tween20 (Sigma-Aldrich) in sterile water (Kankanala et al., 2007). A conidial suspension of *M. oryzae* in 0.025% (v/v) Tween 20 was adjusted to appropriate conidial concentrations using a hemacytometer.

### Plasmid construction and rice transformation

GCaMP6f was cloned into the plant expression vector pGWB554 using standard molecular techniques, as described previously (Choi et al., 2014; Weigand et al., 2021). The resultant construct was transformed into rice cultivar Kitaake using *Agrobacterium tumefaciens* strain LBA4404. Briefly, *35S::GCaMP6f-mCherry* (*GCaMP6fmC*) was delivered into rice calli using *Agrobacterium*-mediated transformation (Hiei et al., 1994). Transformed calli were selected on the half-strength Murashige and Skoog (1/2 MS; Sigma-Aldrich) medium (2.15 g of MS salts, 15 g of sucrose, and 3.5 g of Gelrite [Duchefa Biochemie] in 1 L of water) supplemented with 20 µg·mL^-1^ hygromycin B (Duchefa Biochemie). After root and shoot formation, rice seedlings were transferred to water and acclimated for 2 days. Rice seedlings were then transferred into pots containing Baroker soil (Seoul Bio) and raised in a growth chamber.

### Construction and cloning of the crystal structure of GCaMP6-mCherry

The crystal structures of Ca^2+^-free GCaMP6 (ID: 3wlc), Ca^2+^-bound GCaMP6 (ID: 3wld), and mCherry (ID: 2h5q) were obtained from the RCSB Protein Data Bank (https://www.rcsb.org) (Shu et al., 2006; Ding et al., 2014). These three crystal structures were then used to construct crystal images of GCaMP6-mCherry using the PyMOL software (https://pymol.org) (Ding et al., 2014). The Ca^2+^ sensor construct GCaMP6f-mCherry (GCaMP6fmC) was cloned into the vector pGWB554 under the control of the Cauliflower mosaic virus (CaMV) 35S promoter (**Supplementary Figure 1A**).

### Generation of Ca^2+^ sensor (*35S::GCaMP6fmC)* transgenic rice lines

**Figure 1:**
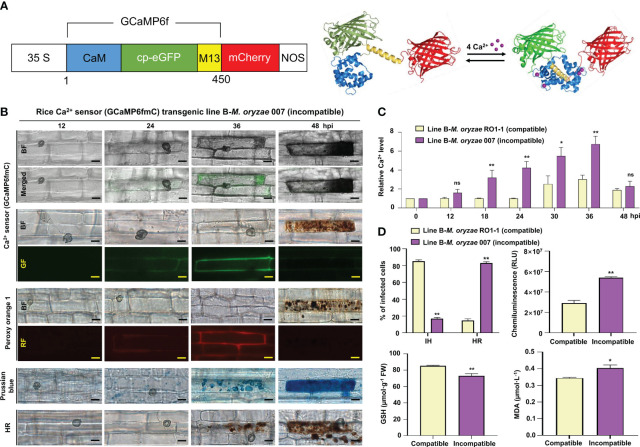
Ca^2+^ influx mediates ROS- and iron-dependent HR cell death in rice immune responses. **(A)** Schematic diagram of the Ca^2+^ sensor construct. When Ca^2+^ is free, the calmodulin (CaM) domain is dissociated from M13; however, the binding of Ca^2+^ to CaM promotes CaM-M13 binding, leading to increased eGFP fluorescence. mCherry (red fluorescence) serves as an internal fluorescence reference. **(B)** Time-course images of Ca^2+^ influx, ROS and Fe^3+^ accumulation, and HR cell death in the leaf sheaths of rice (Kitaake) Ca^2+^ sensor (GCaMP6fmC) transgenic line B during avirulent *M. oryzae* infection. Bars = 10 µm. **(C)** Time-course quantification of Ca^2+^ influx during virulent and avirulent *M. oryzae* infection. At least three regions of interest (ROIs) were selected to quantify Ca^2+^ changes by calculating the GCaMP6f/mCherry ratio at different time points (hpi) using the software ImageJ. The ratio values of GCaMP6f/mCherry were compared with those of the controls (0 hpi). **(D)** Quantification of disease phenotypes (HR/IH) (48 hpi) and ROS (36 hpi), GSH (48 hpi), and lipid (MDA) peroxidation (48 hpi) levels during virulent and avirulent *M. oryzae* infection. Data are represented as the mean ± SD (n = 4 leaf sheaths from different plants). Asterisks indicate statistically significant differences (*P< 0.05, **P< 0.01; Student’s *t*-test). hpi, hours post**-**inoculation; ns, non-significant; IH, invasive hyphae; HR, hypersensitive response; RLU, relative luminescent units; MDA, malondialdehyde.

The seeds of rice cultivar Kitaake and Ca^2+^ sensor (*35S::GCaMP6fmC*) transgenic rice lines were hulled, sterilized first with 100% ethanol for 1 min and then with 50% Clorox for 30 min, and washed three times with 3DW. The surface-sterilized seeds were then cultured on 1/2 MS medium (Sigma-Aldrich, St. Louis, MO) at 25°C under continuous light for 2 weeks. Leaves were collected from rice plants and subjected to genomic DNA extraction using the cetyltrimethylammonium bromide (CTAB) method. Plants were genotyped by PCR as described previously (Kim et al., 2011), and Ca^2+^ sensor (GCaMP6fmC) transgenic lines were identified using the hygromycin gene-specific and GCaMP6f-specific primers (**Supplementary Table 1**). Ca^2+^ sensor (GCaMP6fmC) expression was verified in the transgenic lines by PCR using hygromycin resistance (HygR) and GCaMP6fmC primers (**Supplementary Figure 1B**). Without Ca^2+^ binding, the GCaMP6fmC has low intrinsic fluorescence. However, the green fluorescence of GCaMP6fmC increases after binding of Ca^2+^ to the calmodulin domain (**Figures 1A, B**).

### RNA extraction and gene expression analysis

Total RNA was extracted from rice plants using the TRIzol Reagent (Invitrogen) and used for cDNA synthesis. Transcript levels of rice *Glutathione Reductase 1* (*OsGR1*), *OsGR2*, *OsGR3*, and *Ubiquitin* (*OsUbi*) genes were analyzed by reverse transcription polymerase chain reaction (RT-PCR) and real-time quantitative RT-PCR (real-time qRT-PCR) using gene-specific primers (**Supplementary Table 1**). Transcript levels of *OsGR1–3* were normalized relative to that of *OsUbi* and presented as mean ± standard deviation (SD) of three biological replicates. The experiments were repeated three times.

### *M. oryzae* inoculation and Ca^2+^ influx inhibitor and enhancer treatment of rice

To investigate Ca^2+^-mediated iron- and ROS-dependent ferroptotic cell death in rice during *M. oryzae* infection, Ca^2+^ influx inhibitors, including ethylene glycol-bis(2-aminoethylether)-*N, N, N′, N′*-tetra-acetic acid (EGTA; Sigma-Aldrich) (Atkinson et al., 1990; Cessna and Low, 2001), verapamil hydrochloride (verapamil; Sigma-Aldrich) (Beneloujaephajri et al., 2013), N-acetyl-cysteine (NAC; Sigma-Aldrich) (Sun et al., 2012), neomycin sulfate (neomycin; ChemCruz) (Franklin-Tong et al., 1996), LiCl (Tokyo Chemical Industry) (Moyen et al., 1998), AlCl_3_ (Sigma-Aldrich) (Kadota et al., 2005), and ruthenium red (RR; ChemCruz) (Bae et al., 2003), and Ca^2+^ influx enhancers, including acibenzolar-*S*-methyl (ASM; Sigma-Aldrich) (Brisset et al., 2000; Buonaurio et al., 2002), diamide (Sigma-Aldrich) (Kosower et al., 1969; Gilge et al., 2008), trifluoperazine hydrochloride (TFP; Sigma-Aldrich) (Kang et al., 2017), CaCl_2_/calcimycin (C/C; Sigma-Aldrich) (Verma et al., 2011), CaCl_2_/H_2_O_2_ (C/H; Sigma-Aldrich) (Rentel and Knight, 2004), rotenone (Sigma-Aldrich) (Li et al., 2003), and butylmalonic acid (BMA; Sigma-Aldrich) (Kamga et al., 2010), were applied onto rice leaf sheaths. All Ca^2+^ influx inhibitors and enhancers used in this study were treated on rice leaf sheaths with appropriate concentrations that never inhibited *M. oryzae* spore germination, appressorium formation and growth that are important for *M. oryzae* infection in rice. Among the Ca^2+^ influx enhancers, diamide and BMA were used to induce the depletion of reduced glutathione (GSH) (Gilge et al., 2008; Kamga et al., 2010), and rotenone was used to inhibit ROS burst by mitochondrial complex I (Li et al., 2003). Each Ca^2+^ influx inhibitor was applied onto a 5–7 cm long section of rice leaf sheath, together with avirulent *M. oryzae* 007 (5 × 10^5^ conidia·mL^–1^) inoculation. Similarly, each Ca^2+^ influx enhancer (except TFP and rotenone) was applied onto a 5–7 cm long section of rice leaf sheath, together with virulent *M. oryzae* RO1-1 (5 × 10^5^ conidia·mL^–1^) inoculation; TFP or rotenone was applied to rice sheath (5–7 cm long section) at 23 h post-inoculation (hpi) with virulent *M. oryzae* RO1-1. The Ca^2+^ influx inhibitor/enhancer treated and pathogen-inoculated rice leaf sheaths were incubated at 25°C under moist conditions. Rice leaf sheath samples were collected at different time points after inoculation with *M. oryzae.*

### Determination of infection types

The epidermal cell layers excised from the inoculated rice leaf sheaths were observed under a microscope (Zeiss equipped with Axioplan 2; Campbell, CA), as described previously (Kankanala et al., 2007). The infected epidermal cells were counted and categorized into two types: cells with invasive hyphae (IH) and cells with hypersensitive response (HR) cell death. Infected cells of each infection type were quantified. The experiment was repeated three times.

### Ca^2+^ influx detection and quantification

Images of Ca^2+^ influx in rice sheath cells were taken using a microscope (Zeiss equipped with Axioplan2) with bright field filter, green fluorescence filter (excitation [Ex]/emission [Em] wavelengths: 450–490/515–565 nm), and red fluorescence filter (Ex/Em: 546/590 nm). At least three regions of interest (ROIs) were selected to quantify changes in Ca^2+^ levels by calculating the GCaMP6f/mCherry ratio at different time points or at 36 hpi using the ImageJ software. Green and red fluorescence signal intensities were measured using ImageJ installed with the Macro (Weigand et al., 2021). Data were exported into an Excel file, and the ratio of green to red fluorescence signal intensities was calculated. The values were compared with those of the mock control and expressed as relative fluorescence intensities (RFI). Images were taken using Leica TCS SP5 confocal microscope (Leica, Mannheim, Germany), with bright field, EGFP (Ex/Em: 488/500–540 nm), and mCherry (Ex/Em: 587/600–680 nm) filters, and merged.

### Visualization and fluorescence quantification of cellular Ca^2+^ levels using Ca^2+^ indicator Fluo-5F AM

**Figure 2:**
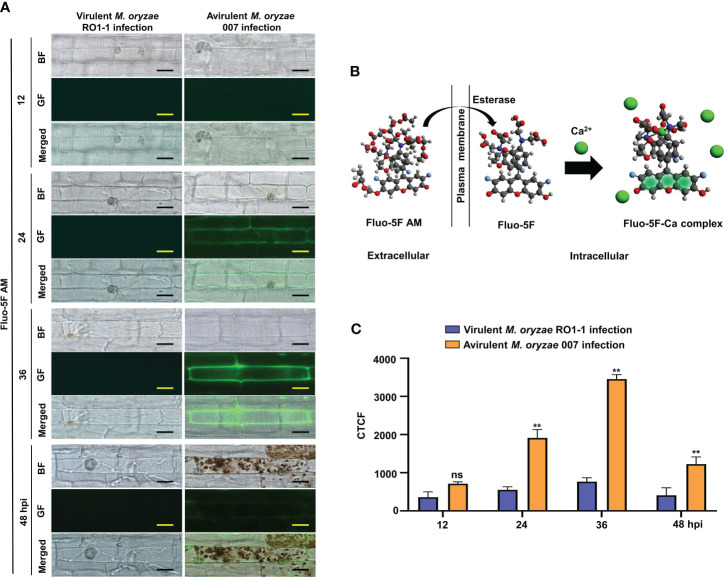
Dynamics of Ca^2+^ influx in the leaf sheaths of rice Kitaake during *Magnaprthe oryzae* 007 (avirulent) and RO1-1 (virulent) infection. **(A)** Time-course images of Ca^2+^ influx in the rice leaf sheaths during *M. oryzae* infection. The rice leaf sheaths were inoculated with *M. oryzae* RO1-1 (virulent) and 007 (avirulent), and the Ca^2+^ dynamics were detected at 12, 24, 36, and 48 hpi. Rice leaf sheath cells were stained with Fluo-5F AM and were observed under a microscope (Zeiss equipped with Axioplan 2) using a bright field filter and/or the green fluorescence filter. Bars = 10 µm. WT, wild type; BF, bright field; GF, green fluorescence. hpi, hours post-inoculation. **(B)** Schematic diagram of Ca^2+^ detection by Fluo-5F AM. When Fluo-5F AM enters the cytoplasm from the apoplast, the AM is cut off by cellular esterases. When free Ca^2+^ binds to Fluo-5F, the Fluo-5F-Ca complex becomes fluorescent. **(C)** Time-course quantification of Ca^2+^ influx during *M. oryzae* infection. At least three regions of interest (ROIs) were selected to quantify Ca^2+^ changes by calculating the corrected total cell fluorescence (CTCF) at different time points (hpi) using the software ImageJ. Data are represented as the mean ± SD (n = 4 leaf sheaths from different plants). Asterisks above bars indicate significantly different means (**P< 0.01; Student’s *t*-test). hpi, hours post-inoculation; ns, non-significant.

Fluo-5F AM (Invitrogen) is a low affinity intracellular Ca^2+^ indicator suitable for detecting high intracellular Ca^2+^ levels ranging from 1 µM to 1 mM. Binding of Ca^2+^ to Fluo-5F is catalyzed by cellular esterases that break the ester bonds of Fluo-5F AM (**Figure 2B**). Intracellular Ca^2+^ dynamics in *M. oryzae*-infected rice sheaths were determined by the Ca^2+^ indicator Fluo-5F AM (Invitrogen) (Eaddy and Schnellmann, 2011). Fluo-5F AM was used to visualize intracellular Ca^2+^ ions during *M. oryzae* infection. Briefly, the epidermal layer recovered from the rice leaf sheaths was immersed in 0.5 M sucrose for 10 min for rapid plasmolysis. The leaf sheath epidermis was then incubated in a solution of Fluo-5F AM at a final concentration of 50 µM at 37°C for 1 h, followed by a rapid washing using 3DW. The epidermis was kept at room temperature for another 30 min to allow the reaction to occur. To visualize both Ca^2+^ and ROS simultaneously inside the same rice cell, a mixture of 50 µM Fluo-5F AM and 5 µM PO1 was used as described above. The above steps were performed under light-blocking conditions. Samples were observed under a fluorescence microscope (Zeiss equipped with an Axioplan 2; Campbell, CA) with a bright field (BF) filter and/or a green fluorescence (GF) filter. Fluorescence intensities of at least three regions of interest (ROIs) were selected to quantify changes in Ca^2+^ levels by measuring green fluorescence intensities at different time points using ImageJ software (Grossi et al., 2016). Corrected total cell fluorescence (CTCF) values were calculated as previously described (Jakic et al., 2017; Bora et al., 2021): CTCF = integrated fluorescence density – (ROI area × mean fluorescence of background readings).

### Detection of apoplastic (intercellular) Ca^2+^ concentration

Rice leaf sheaths were inoculated with avirulent *M. oryzae* 007 (4 × 10^5^ conidia·mL^-1^) in 10 mM EGTA, and apoplastic washing fluid was prepared at 12, 24, 36, and 48 hpi as described previously (Rohringer et al., 1983), with some modifications. The *M. oryzae*-inoculated and/or EGTA-treated rice leaf sheaths were vacuum-infiltrated with 3DW (distilled water) and then centrifuged at 4,000× *g*. The supernatants were completely evaporated to dryness using an evaporator, and the residue was resuspended in a certain amount of 3DW. Apoplastic Ca^2+^ concentration in rice leaf sheaths was measured using the *o*-CPC method (Corns and Ludman, 1987), which is based on the reaction of Ca^2+^ with *o*-cresolphthalein complexone (*o*-CPC), resulting in the formation of an intense violet-colored complex. Briefly, 100 μL of intercellular fluid prepared from rice leaf sheath was added to a reaction solution containing 375 μL of 1 M ethanolamine (pH 10.6; Sigma-Aldrich), 71.6 μL of 100 mM 8-hydroxyquinoline (Sigma-Aldrich), 8.2 μL of 10 mM *o*-CPC (Sigma-Aldrich), 2.32 μL of 37% HCl (Samchun), and 3DW (up to a total volume of 1 mL). The absorbance of the Ca^2+^-*o*-CPC complex was measured at 575 nm using SP-2000 UV spectrophotometer (SmartPlus) (Corns and Ludman, 1987). Apoplastic Ca^2+^ concentration in rice leaf sheaths was calculated based on a standard curve obtained using 0–25 μM standard CaCl_2_ (Sigma-Aldrich).

### ROS detection and quantification

ROS (H_2_O_2_) localization in *M. oryzae*-infected rice sheath cells was determined by Peroxy Orange 1 (PO1; Sigma-Aldrich) staining. The red fluorescent ROS indicator PO1 simultaneously visualizes Ca^2+^ (green fluorescence) and ROS accumulation inside the same infected rice cell through its distinct red fluorescence emission. Briefly, the epidermal cell layer peeled off from rice leaf sheaths was soaked in 5 µM PO1 for 40 min in the dark at room temperature (Muhlemann et al., 2018; Samuel et al., 2022). The samples were washed three times with 1× phosphate-buffered saline (PBS) and observed under a fluorescence microscope (Zeiss equipped with Axioplan 2; Campbell, CA) (Ex/Em: 546/590 nm).

A chemiluminescence assay (Singh et al., 2016; Chen et al., 2022) was performed to measure ROS levels in *M. oryzae*-infected rice leaf sheath cells. Briefly, small sections of epidermal cell layer (0.5 cm × 0.2 cm) were transferred into individual wells of a 96-well plate, with each well containing 100 µL of chemiluminescent solution (30 µL of luminol [Bio-Rad, Hercules, CA], 1 µL of 1 mg·mL^–1^ horseradish peroxidase [HRP; Jackson Immunoresearch, West Grove, PA], and 69 µL of Milli-Q water), and incubated in the dark for 5 min at room temperature. ROS chemiluminescence was detected using GloMax 96 Microplate Luminometer (Promega, Madison, WI) and expressed as relative luminescence units (RLU). Experiments were independently repeated three times.

### Ferric ion detection

Fe^3+^ accumulation in rice leaf sheath cells was detected and visualized by Prussian blue staining (Liu et al., 2007). Briefly, the epidermal layer of rice leaf sheath cells was isolated and then stained with a Prussian blue solution (7% potassium ferrocyanide [Sigma-Aldrich] and 2% HCl [Samchun], v:v = 1:1) for 15 h at room temperature and washed three times with 3DW. The stained epidermal cells were observed under a microscope (Zeiss equipped with Axioplan 2, Campbell, CA). Fe^3+^ were detected as a bright blue signal in sheath epidermal cells, because of their binding to ferric ferrocyanide in cells.

### Lipid peroxidation (MDA) assay

Lipid peroxidation in rice leaf sheath samples was determined by measuring the level of malondialdehyde (MDA), a product of unsaturated fatty acid peroxidation, using thiobarbituric acid (TBA). Rice leaf sheath tissue was ground in liquid nitrogen, and the powdered tissue was mixed with an equal amount of reaction solution (0.5% [w/v] TBA [Sigma-Aldrich], 20% [v/v] trichloroacetic acid [TCA; Sigma-Aldrich], and 0.25 mL of 175 mM NaCl in a total of 2 mL of 50 mM Tris-Cl [pH 8.0]). Samples were incubated in boiling water for 5 min and then centrifuged at 14,000 × *g* for 5 min at 4°C. The absorbance (optical density [OD]) of each supernatant was measured at 450, 532, and 600 nm with the SP-2000UV spectrophotometer (Woongki Science, Seoul), as previously described (Zhang et al., 2009). MDA concentration was calculated using the following equation (Dangol et al., 2019).


$$C_{MDA}=[6.45\times (OD_{532}-OD_{600})]-(0.56\times OD_{450})$$

where *C_MDA_* is the concentration of MDA, and *OD_450_*, *OD_532_*, and *OD_600_* represent the OD of the supernatant at 450, 532, and 600 nm, respectively.

### Measurement of GSH levels

The content of reduced glutathione (GSH) in rice leaf sheaths was measured spectrophotometrically. Freshly harvested conidial suspensions (4 × 10^5^ conidia·mL^-1^) of *M. oryzae* strains, with or without Ca^2+^ influx inhibitor or enhancer, were used to inoculate rice leaf sheaths. The inoculated leaf sheaths were incubated for 48 h in the dark in a moistened box at 25°C and then ground in liquid nitrogen. Equal amounts of the powdered sample and 5% (w/v) metaphosphoric acid (Sigma-Aldrich) were mixed, and the homogenates were centrifuged at 21,000 × *g* for 20 min at 4°C. The supernatants were collected, and each supernatant was passed through a 0.45 µm nylon filter (Sigma-Aldrich). GSH quantification was performed as described previously (Griffith, 1980; Airaki et al., 2011). Briefly, 100 µL of the filtered supernatant was added to 600 µL of reaction buffer (100 mM sodium phosphate buffer [pH 7.5] and 1 mM EDTA [Sigma-Aldrich]). Then, 40 µL of 0.4% (w/v) 5,5’-dithiobis (2-nitrobenzoic acid) (DTNB, Sigma-Aldrich) and 350 µL of Milli-Q water were added to each sample, and the mixtures were incubated at room temperature for 5 min. To detect GSH, the absorbance of each mixture was measured at a wavelength of 412 nm using a spectrophotometer. The GSH content of each rice leaf sheath sample was quantified by constructing a calibration curve using a wide range of concentrations (0–25 µM) of standard GSH (Sigma-Aldrich).

### Analysis of the inhibitory effect of Ca^2+^ on glutathione reductase activity *in vitro*

To investigate the effect of Ca^2+^ influx on the conversion of glutathione disulfide (GSSG; oxidized glutathione) into GSH (reduced glutathione) by GR, 30 µL of 1 mM GSSG (Sigma-Aldrich) and 20 µL of 4.8 mM NADPH (nicotinamide adenine dinucleotide phosphate, reduced form; Sigma-Aldrich) were added to 50 mM Tris-Cl (pH 7.5). Then, 10 µL (0.06 U) of rice GR (Koma Biotech) and yeast GR (Sigma-Aldrich) each was added to the mixture to initiate the reduction of GSSG, and the total volume of the reaction mixture was increased to 1 mL. According to the Beer-Lambert Law, 0.1 mM NADPH has an optical density which is equal to 0.622 through a 1 cm light path. Therefore, because of the consumption of NADPH during the reduction of GSSG, the OD_340_ of the sample was expected to decrease with a molar extinction coefficient of 6.22 mmol^-1^·cm^-1^ at 340 nm. The decrease in the absorbance of each sample was measured at 340 nm using SP-2000 UV spectrophotometer (SmartPlus). The reactions were monitored for 5 min at room temperature.

To perform the inhibition assay, different volumes of 100 mM CaCl_2_ were added to the reaction system so that the final concentrations of CaCl_2_ were 0, 10, 20, 30, and 50 mM. To investigate the inhibitory effect of different cations on GSSG reduction *in vitro*, 20 mM CaCl_2_ (Sigma-Aldrich), MgCl_2_ (Sigma-Aldrich), NaCl (Samchun), and KCl (Duksan) were added to the reaction system. The reaction was then monitored for 5 min by detecting the decrease in OD at 340 nm.

### Data analysis

All the results are expressed as the mean ± standard deviation (SD). Statistical comparisons were performed by the least significant difference (LSD) test and Student’s *t*-test using GraphPad Prism 8 software (GraphPad Software, Inc., San Diego, CA, USA).

## Results

### Ca^2+^ influx mediates ROS- and iron-dependent HR cell death in rice-*M. oryzae* interactions

**Figure 3:**
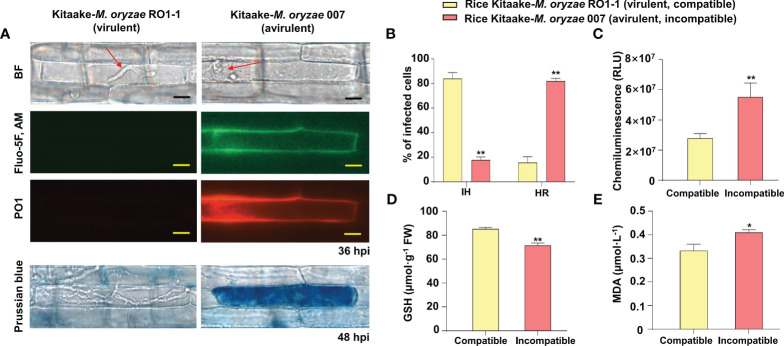
Images and quantification of Ca^2+^ influx, ROS and iron accumulation, hypersensitive response (HR) cell death, reduced glutathione (GSH) depletion, and lipid peroxidation in rice leaf sheaths during *Magnaporthe oryzae* infection. **(A)** Images of Ca^2+^ influx and ROS and iron accumulation in rice leaf sheaths during *M. oryzae* infection. The leaf sheaths of Kitaake (WT) were inoculated with *M. oryzae* RO1-1 (virulent) and 007 (avirulent). Rice leaf sheath cells were stained with a mixture of Fluo-5F AM and Peroxy Orange 1 (PO1) to visualize Ca^2+^ influx and H_2_O_2_ accumulation in the rice cell during infection. Fe^3+^ accumulation was detected by Prussian blue staining. Images of Ca^2+^ influx, H_2_O_2_ accumulation, and Fe^3+^ accumulation in rice leaf sheaths were observed under a microscope (Zeiss equipped with Axioplan 2) using a bright field filter and/or fluorescence filters. Bars = 10 µm. Red arrows indicate invasive hyphae (IH). WT, wild type; BF, bright field. **(B)** Quantification of infection phenotypes, classified as cells showing invasive hyphal (IH) growth and HR cell death, in the leaf sheaths (48 hpi). **(C)** ROS quantification (36 hpi) in rice cells via a chemiluminescence assay using GloMax 96 Microplate Luminometer (Promega, Madison, WI). Values are mean ± standard deviation (SD; *n* = 10) of the total relative luminescence units (RLU) of different rice sheath discs. **(D)** Quantification of GSH levels in rice leaf sheaths (48 hpi). Values represent mean ± SD (*n* = 4) of GSH concentrations in the leaf sheaths of different plants. GSH reacts with 5,5’-dithiobis (2-nitrobenzoic acid) to generate 2-nitro-5-thiobenzoic acid, which was measured using the SP-2000UV spectrophotometer (Woongki Science, Seoul) at a wavelength of 412 nm. **(E)** Quantification of lipid peroxidation in rice leaf sheaths (48 hpi) by measuring malondialdehyde (MDA) levels. Values are mean ± SD (*n* = 4) of MDA concentrations in the leaf sheaths of different plants. Asterisks above bars indicate significantly different means (*P< 0.05, **P< 0.01; Student’s *t*-test). Experiments were repeated three times with similar results. hpi, hours post-inoculation.

We first examined time-course images and levels of Ca^2+^ influx in the leaf sheaths of rice Kitaake during *M. oryzae* RO1-1 (virulent) and 007 (avirulent) infection (**Figure 2**). During virulent (compatible) *M. oryzae* RO1-1 infection, primary hyphae grew from the appressorium, differentiated into thicker, bulbous invasive hyphae (IH) in the invaded rice cell to spread into neighboring cells (**Figure 2A**). However, avirulent (incompatible) *M. oryzae* 007 infection induced severe HR death response with dark brown cellular aggregates at 48 h post-inoculation (hpi) in rice leaf sheath cells. We have used the Ca^2+^ indicator Fluo-5F AM to monitor time-course changes in cytoplasmic Ca^2+^ influx by visualizing and quantifying Ca^2+^ fluorophores in living rice cells (Eaddy and Schnellmann, 2011). Fluo-5F is initially non-fluorescent. However, it becomes fluorescent when it binds to free Ca^2+^ in cells. During avirulent *M. oryzae* 007 infection, cytoplasmic Ca^2+^ influx began to appear at 12 hpi, increased markedly at 24 hpi, and peaked at 36 hpi (**Figures 2A, C**). At 48 hpi, extensive HR cell death occurred with dark brown cellular aggregates; however, Ca^2+^ influx levels decreased rapidly. By contrast, cytoplasmic Ca^2+^ levels were rarely or not detected in rice cells during virulent *M. oryzae* RO1-1 infection. We also stained rice leaf sheaths with a mixture of Fluo-5F AM and Peroxy Orange 1 (PO1) to visualize localization of Ca^2+^ and ROS (H_2_O_2_) accumulation (36 hpi) during infection, respectively (**Figure 3A**). The interplay of Ca^2+^ and ROS during plant immunity is a well-known phenomenon, but its mechanisms are not fully understood. Interestingly, we observed a marked co-localization of Ca^2+^ and ROS accumulation around invasive hyphae (IH) as well as inside the invaded and neighboring cells of rice leaf sheaths at 36 hpi with avirulent *M. oryzae* 007 (**Figure 3A**). To determine the role of ferric ions (Fe^3+^) in HR cell death during *M. oryzae* infection, we further stained rice leaf sheath tissues with Prussian blue solution to detect Fe^3+^, as described previously (Dangol et al., 2019). Unlike Ca^2+^ or ROS accumulation, Fe^3+^ was observed (blue color) at hyphal invasion sites in the HR cell death response of rice leaf sheath cells at 48 hpi with avirulent *M. oryzae* 007 (**Figure 3A**). By contrast, Ca^2+^, ROS and iron (Fe^3+^) accumulation was not detected in the rice sheath cells infected by virulent *M. oryzae* RO1-1 (**Figure 3A**). Avirulent *M. oryzae* 007 infection, but not virulent *M. oryzae* RO1-1 infection, significantly induced Ca^2+^ and ROS accumulation (36 hpi), iron accumulation (48 hpi), HR cell death (48 hpi), reduced glutathione (GSH, γ-L-glutamyl-L-cysteinylglycine) depletion (48 hpi), and lipid peroxidation (48 hpi) in rice leaf sheath cells (**Figures 3A, D**).

The Ca^2+^ sensor was designed as the mCherry fused to the N-terminus of GCaMP6f and consisted of two tandem fluorescent proteins (Weigand et al., 2021). The protein-based Ca^2+^ sensor (GCaMP6fmC) was used as an alternative tool to compare two separate cytoplasmic Ca^2+^ influxes in the wild type and Ca^2+^ sensor transgenic rice lines. To investigate if Ca^2+^ mediates ferroptotic cell death in rice immunity, we next generated Ca^2+^ sensor rice (cultivar Kitaake) transgenic lines as an alternative Ca^2+^ visualization tool, in which GCaMP6f-mCherry fusion Ca^2+^ reporter (GCaMP6fmC) can be expressed to emit strong green fluorescence by binding of Ca^2+^ to the calmodulin domain (**Figure 1A**; **Supplementary Figure 1**). The Ca^2+^ sensor (GCaMP6fmC) made it possible to specifically detect and visualize intracellular Ca^2+^ changes inside living rice cells without any additional calcium indicator staining. Avirulent (incompatible) *M. oryzae* 007 grew poorly, with only a few invasive hyphae (IH) leading to HR cell death in rice leaf sheaths (**Figures 2A**, **1B, D**). Ca^2+^ influx levels in the rice leaf sheaths were measured by confocal and fluorescence microscopy (**Figure 2A**, green fluorescence in the GF images; **Figure 1B**; **Supplementary Figures 1C**, **2**). Peroxy Orange 1 and Prussian blue staining showed the accumulation of ROS (H_2_O_2_) and Fe^3+^, respectively, in leaf sheath cells (**Figure 1B**, red fluorescence in the RF images for ROS and blue-colored deposits for Fe^3+^). Ca^2+^ and ROS accumulation was significantly induced around the IH and within the invaded rice cells at 36-48 hpi with avirulent *M. oryzae* 007 (**Figures 1B, D**). By contrast, ROS accumulation was not detected in rice cells infected with virulent *M. oryzae* RO1-1. Avirulent *M. oryzae* 007, but not virulent *M. oryzae* RO1-1, significantly induced Fe^3+^ accumulation inside and around the IH in rice sheath cells at 36-48 hpi (**Figure 1B**). Fe^3+^ was observed as a blue color inside the IH and at the hyphal invasion sites in the HR cell death response where ROS accumulated. HR cell death was detected as vesicle-containing dark brown cellular aggregates inside the infected rice cells at 48 hpi (**Figure 1B**). During avirulent *M. oryzae* infection, abundant ROS bursts and HR cell death at 48 hpi were accompanied by significant depletion of reduced glutathione (GSH, γ-L-glutamyl-L-cysteinylglycine) and significant increase in lipid peroxidation [malondialdehyde (MDA) levels] (**Figures 1B, D**). GSH is one of the major water-soluble small molecule antioxidants that protect plant cells from oxidative damage (Airaki et al., 2011).

Ca^2+^ influx into the leaf sheath cells of Ca^2+^ sensor (GCaMP6fmC) transgenic line B was quantified over time during *M. oryzae* RO1-1 (virulent) and 007 (avirulent) infection (**Figure 1C**). During avirulent *M. oryzae* 007 infection, we observed *M. oryzae* appressorium formation at 12 hpi, hyphal invasion initiation at 24 hpi, strong Ca^2+^ influx at 18-36 hpi, abundant HR cell death at 48 hpi, along with ROS and iron accumulation, GSH depletion, and lipid peroxidation (**Figures 1B, D**). Overall, our data suggest that avirulent *M. oryzae* 007 infection induces higher levels of Ca^2+^ influx to mediate ROS- and iron-dependent ferroptotic cell death compared with virulent *M. oryzae* RO1-1 infection (**Figures 2**-**1**; **Supplementary Figure 2**).

### The calcium chelator EGTA effectively blocks Ca^2+^-mediated ROS- and iron-dependent HR cell death

**Figure 4:**
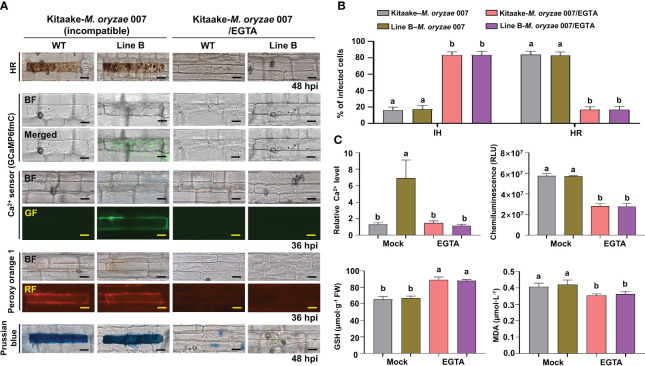
EGTA suppresses Ca^2+^-mediated ROS- and iron-dependent HR cell death in rice immune responses. **(A)** Images of HR cell death (48 hpi), Ca^2+^ influx (36 hpi), ROS accumulation (36 hpi), and Fe^3+^ accumulation (48 hpi) in the leaf sheaths of rice Kitaake (WT) and Ca^2+^ sensor (GCaMP6fmC) transgenic line B during avirulent *M. oryzae* 007 infection after 10 mM EGTA treatment. Bars = 10 µm. **(B)** Quantification of IH/HR infection phenotypes (48 hpi). **(C)** Quantification of Ca^2+^ influx (36 hpi), ROS accumulation (36 hpi), reduced glutathione (GSH) levels (48 hpi), and lipid peroxidation (MDA) levels (48 hpi). For Ca^2+^ quantification, at least three ROIs were selected to calculate the GCaMP6f/mCherry ratio at 36 hpi using the software ImageJ. The ratio values of GCaMP6f/mCherry were compared with that of the control (Mock, Kitaake-*M. oryzae* 007). Data are represented as the mean ± SD (*n* = 4 leaf sheaths from different plants). Different letters above the bars indicate significantly different means, as determined by the least significant difference (LSD) test (P< 0.05). WT, wild type (Kitaake); EGTA, ethylene glycol-bis(2-aminoethylether)-*N, N, N′, N′*-tetra-acetic acid; hpi, hours post-inoculation; IH, invasive hyphae; HR, hypersensitive response; RLU, relative luminescent units; MDA, malondialdehyde.

During avirulent *M. oryzae* 007 infection, Ca^2+^ *-*mediated ROS and iron accumulation in cells induced ferroptotic HR cell death (**Figure 4**). Avirulent *M. oryzae* infection induced high levels of Ca^2+^ influx and ROS and iron accumulation in rice leaf sheath cells, as visualized by confocal and fluorescence microscopy (**Figure 4A**). We applied the membrane-nonpermeable calcium chelator ethylene glycol-bis(2-aminoethylether)-*N, N, N′, N′*-tetra-acetic acid (EGTA) (Ellis-Davies and Kaplan, 1994) onto rice leaf sheaths to investigate whether EGTA blocks Ca^2+^ influx from the apoplast to the cytoplasm during rice HR cell death. EGTA (10 mM) treatment inhibited the accumulation of Ca^2+^, ROS and iron, and the induction of HR cell death by avirulent *M. oryzae* infection, which ultimately allowed the fungal hyphae to grow normally inside the leaf sheath cells (**Figure 4A**). We also quantified the infection phenotypes (IH and HR), Ca^2+^ influx, ROS production, and GSH and MDA levels in mock (water)- and EGTA-treated rice Kitaake and Ca^2+^ sensor (GCaMP6fmC) transgenic line B at different times after inoculation with avirulent *M. oryzae* 007 (**Figures 4B, C**). EGTA-treated leaf sheaths contained more pathogen-infected cells but fewer HR cells, compared to the mock (water)-treated leaf sheaths during avirulent *M. oryzae* 007 infection (**Figure 4B**). EGTA treatment significantly inhibited Ca^2+^ influx and ROS production, correlated with strong GSH production and reduced lipid peroxidation in avirulent *M. oryzae* 007-infected rice cells (**Figure 4C**).

**Figure 5:**
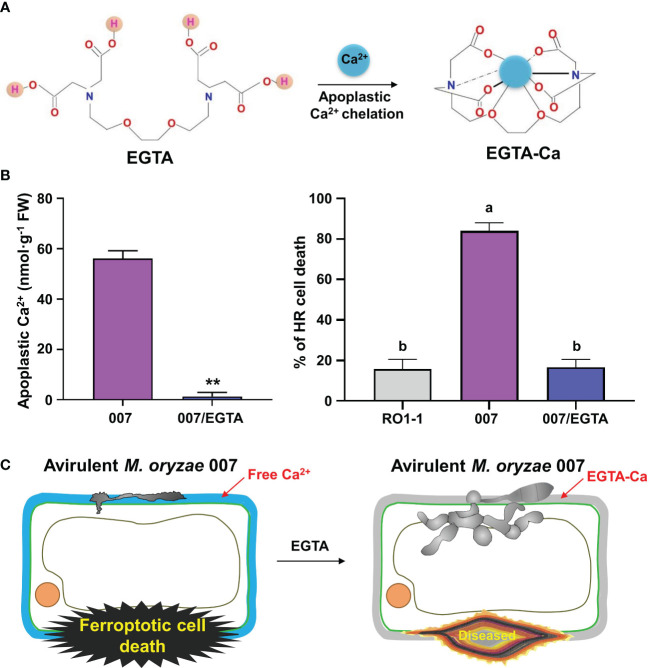
Ca^2+^ chelation by EGTA suppresses ferroptotic cell death in rice leaf sheaths during avirulent *Magnaporthe oryzae* 007 infection. **(A)** Schematic diagram of apoplastic Ca^2+^ chelation by EGTA. The binding of Ca^2+^ to EGTA forms the EGTA-Ca complex. **(B)** Quantification of apoplastic Ca^2+^ concentrations and HR cell death in rice (Kitaake) leaf sheaths treated with 10 mM EGTA during *M. oryzae* RO1-1 (virulent) and 007 (avirulent) infection. Data are represented as the mean ± SD (*n* = 4 leaf sheaths from different plants). Asterisks indicate significantly different means (**P< 0.01; Student’s *t*-test). Different letters above the bars indicate significantly different means, as determined by the least significant difference (LSD) test (*P*< 0.05). Experiments were repeated three times with similar results. **(C)** Schematic diagram of inhibition of ferroptotic cell death by apoplastic Ca^2+^ chelation using EGTA. During avirulent *M. oryzae* 007 infection, EGTA treatment effectively inhibits apoplastic Ca^2+^ influx into the cytoplasm to completely attenuate ferroptotic HR cell death in rice sheaths, which ultimately leads to the formation of normal hyphal structures and susceptible disease.

Binding of Ca^2+^ to EGTA forms the EGTA-Ca complex (**Figure 5A**). To investigate if EGTA causes chelation of apoplastic Ca^2+^ in rice cells, we prepared apoplastic washing fluid from *M. oryzae*-inoculated and/or EGTA-treated rice leaf sheaths (**Figure 5**; **Supplementary Figure 3**). Ca^2+^ in the intercellular (apoplastic) fluid of rice leaf sheaths was detected using the calcium-*o*-cresolphthalein complexone (*o*-CPC) method (**Supplementary Figure 3A**). EGTA treatment markedly lowered apoplastic Ca^2+^ levels to block Ca^2+^ influx into the cytoplasm of rice leaf sheaths throughout avirulent *M. oryzae* 007 infection, ultimately suppressing ferroptotic HR cell death, but leading to normal fungal growth and susceptible disease (**Figures 5B, C**; **Supplementary Figure 3B**). Avirulent *M. oryzae* 007 infection resulted in 83.93% of HR cell death in rice sheaths. However, EGTA treatment significantly reduced HR cell death to 16.60%, which is comparable to virulent *M. oryzae* RO1-1 infection (**Figure 5C**). Together, our data suggest that the membrane-nonpermeable calcium chelator EGTA effectively blocks apoplastic Ca^2+^ influx into the cytoplasm to inhibit Ca^2+^-mediated iron-dependent ROS accumulation leading to the formation of normal *M. oryzae* hyphal structure and susceptible blast disease.

### The Ca^2+^ influx enhancer ASM effectively induces Ca^2+^-mediated ROS- and iron-dependent HR cell death

**Figure 6:**
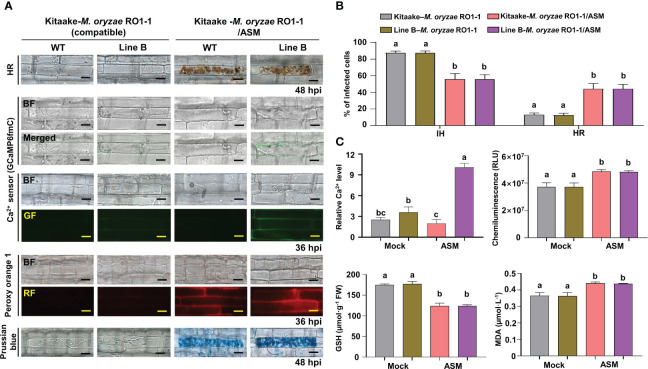
ASM induces Ca^2+^-mediated ROS- and iron-dependent HR cell death in rice immune responses. **(A)** Images of HR cell death (48 hpi), Ca^2+^ influx (36 hpi), ROS accumulation (36 hpi), and ferric ion accumulation (48 hpi) in the leaf sheaths of rice Kitaake (WT) and Ca^2+^ sensor (GCaMP6fmC) transgenic line B during virulent *M. oryzae* RO1-1 infection after 125 µM acibenzolar-*S*-methyl (ASM) treatment. Bars = 10 µm. **(B)** Quantification of IH/HR infection phenotypes (48 hpi). **(C)** Quantification of Ca^2+^ influx (36 hpi), ROS accumulation (36 hpi), reduced glutathione (GSH) levels (48 hpi), and lipid peroxidation (MDA) levels (48 hpi). For Ca^2+^ quantification, at least three ROIs were selected to calculate the GCaMP6f/mCherry ratio at 36 hpi using the software ImageJ. The ratio values of GCaMP6f/mCherry were compared with that of the control (Mock, Kitaake-*M. oryzae* RO1-1). Data are represented as the mean ± SD (*n* = 4 leaf sheaths from different plants). Different letters above the bars indicate significantly different means, as determined by the least significant difference (LSD) test (P< 0.05). WT, wild type; hpi, hours post-inoculation; IH, invasive hyphae; HR, hypersensitive response; RLU, relative luminescent units; MDA, malondialdehyde.

Acibenzolar-*S*-methyl (ASM) is one of the most effective plant activators that can induce systemic acquired resistance (SAR) against a broad range of plant pathogens (Matsuo et al., 2019). During virulent *M. oryzae* RO1-1 infection, fungal hyphae grew well inside the rice leaf sheath epidermal cells (**Figure 6A**). However, compared with the mock (water) treatment, ASM treatment effectively induced HR cell death and ROS and iron accumulation in virulent *M. oryzae*-infected rice cells (**Figures 6A, B**). ASM treatment reduced the number of cells with IH, but increased the number of cells with HR, in susceptible rice leaf sheaths infected (**Figure 6B**). ROS and iron accumulation in rice cells was detected by Peroxy Orange 1 and Prussian blue staining, respectively (**Figure 6A**). We also visualized Ca^2+^ influx under confocal and fluorescence microscopy (**Figure 6A**). The quantification of Ca^2+^ influx revealed that ASM treatment induced Ca^2+^ influx compared with the mock treatment (**Figure 6C**). A chemiluminescent assay showed that ASM treatment significantly induced the accumulation of ROS in rice leaf sheath cells at 36 hpi (**Figure 6C**). However, ASM treatment inhibited GSH accumulation, but enhanced lipid peroxidation (MDA levels), in rice leaf sheath cells at 48 h after inoculation with virulent *M. oryzae* RO1-1 (**Figure 6C**). Collectively, these results suggest that ROS and iron accumulation, GSH depletion and lipid peroxidation are required for ASM-induced Ca^2+^-mediated ferroptotic cell death during rice-*M. oryzae* interactions.

### Different Ca^2+^ influx inhibitors effectively limit Ca^2+^-mediated iron- and ROS-dependent ferroptotic cell death

**Figure 7:**
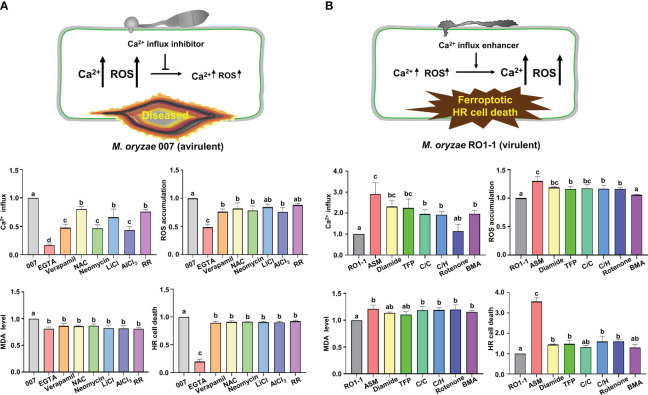
Ca^2+^ influx regulators differentially control Ca^2+^-mediated iron-and ROS-dependent ferroptotic cell death in rice immune responses. **(A, B)** Quantifications of Ca^2+^ influx (36 hpi), ROS accumulation (36 hpi), lipid peroxidation (MDA levels) (48 hpi), and HR cell death (48 hpi) in the leaf sheath cells of rice (Kitaake) Ca^2+^ sensor (GCaMP6fmC) transgenic line B infected with *M. oryzae* 007 (avirulent) and *M. oryzae* RO1-1 (virulent) and treated with Ca^2+^ influx inhibitors. **(A)** Ca^2+^ influx inhibitors included ethylene glycol-bis(2-aminoethylether)-*N, N, N′, N′*-tetra-acetic acid (EGTA), verapamil hydrochloride (verapamil), N-acetyl-cysteine (NAC), neomycin sulfate (neomycin), lithium chloride (LiCl), aluminum chloride (AlCl_3_) and ruthenium red (RR). **(B)** Ca^2+^ influx enhancers included acibenzolar-*S*-methyl (ASM), diamide, trifluoperazine dihydrochloride (TFP), CaCl_2_/calcimycin (C/C), CaCl_2_/H_2_O_2_ (C/H), rotenone and butylmalonic acid (BMA). For Ca^2+^ quantification, at least three ROIs were selected to calculate the GCaMP6f/mCherry ratio at 36 hpi using the software ImageJ. The ratio values of Ca^2+^ influx regulators were compared with those of the controls (*M. oryzae* 007 or *M. oryzae* RO1-1). Data are represented as the mean ± SD (*n*=4 leaf sheaths from different plants). Different letters above the bars indicate significantly different means, as determined by the LSD test (*P<* 0.05). hpi, hours post- inoculation; MDA, malondialdehyde.

Based on the knowledge of pharmacological Ca^2+^ channel modulation identified in animal systems, we selected some cytoplasmic Ca^2+^ influx regulators to investigate whether exogenous chemicals control Ca^2+^-mediated iron- and ROS-dependent ferroptotic cell death in the rice immune response. Different Ca^2+^ influx inhibitors, including EGTA (Atkinson et al., 1990; Cessna and Low, 2001), verapamil hydrochloride (verapamil) (Beneloujaephajri et al., 2013), N-acetyl-cysteine (NAC) (Sun et al., 2012), neomycin sulfate (neomycin) (Franklin-Tong et al., 1996), lithium chloride (LiCl) (Moyen et al., 1998), aluminum chloride (AlCl_3_) (Kadota et al., 2005), and ruthenium red (RR) (Bae et al., 2003) were applied onto the rice leaf sheaths infected with avirulent *M. oryzae* 007 to compare their effects on Ca^2+^ influx, ROS accumulation, lipid peroxidation, and HR cell death (**Figures 4**, **7A**; **Supplementary Figure 4**).

**Figure 8:**
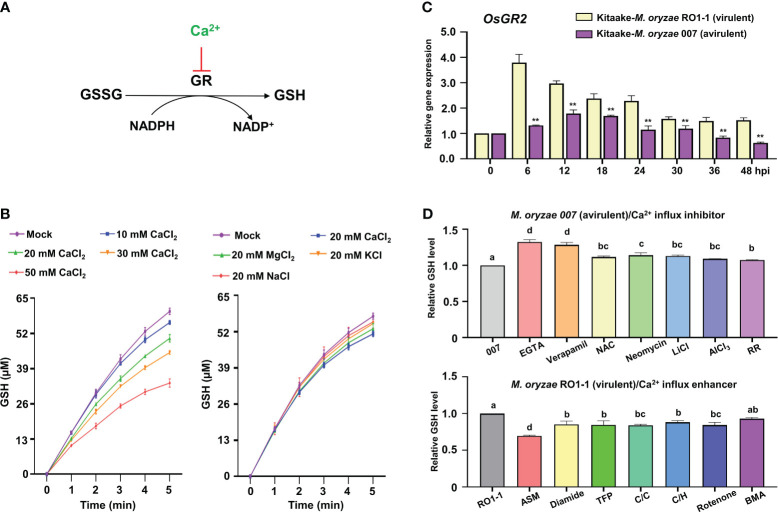
Ca^2+^ influx triggers reduced *OsGR* expression and GSH depletion during *Magnaporthe oryzae* infection. **(A)** Ca^2+^ inhibits GSSG reduction to GSH by glutathione reductase (GR). **(B)** Effects of CaCl_2_, MgCl_2_, KCl, and NaCl on the conversion of GSSG to GSH by rice GR. **(C)** Relative expression of rice *Glutathione Reductase 2* (*OsGR2*) in the leaf sheaths of rice (Kitaake) during *M. oryzae* RO1-1 (virulent) and *M. oryzae* 007 (avirulent) infection. **(D)** Comparison of GSH levels in rice leaf sheaths treated with different Ca^2+^ influx regulators during *M. oryzae* infection. The ratio values of Ca^2+^ influx regulators were compared with those of the controls (*M. oryzae* 007 or *M. oryzae* RO1-1). Data are represented as the mean ± SD (*n* = 4 leaf sheaths from different plants). Asterisks indicate significantly different means (**P< 0.01; Student’s *t*-test). Different letters above the bars indicate significantly different means, as determined by the LSD test (*P*< 0.05). hpi, hours post-inoculation; GSSG, glutathione disulfide; GSH, reduced glutathione.

Treatment with the calcium chelator EGTA dramatically inhibited Ca^2+^ influx, ROS and iron accumulation and HR cell death in rice cells compared with the other tested Ca^2+^ influx inhibitors during avirulent *M. oryzae* infection (**Figures 4**, **7A**). However, other Ca^2+^ influx inhibitors showed variation in the suppression of Ca^2+^ influx, ROS accumulation, lipid peroxidation (MDA level) and HR cell death (**Figure 7A**; **Supplementary Figure 4**). Verapamil treatment is known to reduce cytoplasmic Ca^2+^ levels with attenuated ROS bursts in plant cells (Beneloujaephajri et al., 2013). Inhibition of Ca^2+^ influx by verapamil reduced cytoplasmic Ca^2+^ and ROS accumulation, lipid peroxidation and HR ferroptotic cell death, as detected by Prussian blue staining (**Figure 7A**; **Supplementary Figure 4**). NAC treatment increased GSH concentration and consequently reduced ROS accumulation, lipid peroxidation, and HR cell death, accompanied by decreased Ca^2+^ levels and increased *M. oryzae* infection (**Figures 7A**, **8D**; **Supplementary Figure 4**). LiCl inhibits the release of intracellular Ca^2+^ from vacuoles (Moyen et al., 1998). The two-pore channel 1 (TPC1) channel family is a ROS-responsive Ca^2+^ channel, and aluminum is a specific blocker for TPC1, a voltage-dependent Ca^2+^ permeable channel (Kawano et al., 2004). TPC1 plays an important role in inducing ROS-dependent cytoplasmic Ca^2+^ influx during oxidative stress (Kadota et al., 2005). Neomycin, LiCl, AlCl_3_, and RR significantly inhibited Ca^2+^ influx during avirulent *M. oryzae* 007 infection (**Figure 7A**; **Supplementary Figure 4**). They also significantly reduced the ROS accumulation during avirulent *M. oryzae* 007 infection (**Figure 7A**; **Supplementary Figure 4**). They were relatively effective in limiting lipid peroxidation (MDA level) and HR cell death (**Figure 7A**; **Supplementary Figure 4**). The Ca^2+^ influx inhibitors also suppressed Fe^3+^ accumulation and HR cell death induced by avirulent *M. oryzae* 007 infection, which led to the successful colonization of IH as detected by Prussian blue staining (**Supplementary Figure 4**). These data indicate that different Ca^2+^ influx inhibitors significantly limit Ca^2+^-mediated ROS and iron accumulation, and lipid peroxidation, allowing the formation of normal hyphal structures of avirulent *M. oryzae* 007 in rice leaf sheaths and leading to blast disease (**Figures 4**, **7A**; **Supplementary Figure 4**).

### Different Ca^2+^ influx enhancers effectively trigger Ca^2+^-mediated iron- and ROS-dependent ferroptotic cell death

Different Ca^2+^ influx enhancers, including ASM (Brisset et al., 2000; Buonaurio et al., 2002), diamide (Kosower et al., 1969; Gilge et al., 2008), trifluoperazine dihydrochloride (TFP) (Kang et al., 2017), CaCl_2_/calcimycin (C/C) (Verma et al., 2011), CaCl_2_/H_2_O_2_ (C/H) (Rentel and Knight, 2004), rotenone (Li et al., 2003), and butylmalonic acid (BMA) (Kamga et al., 2010) were applied onto rice leaf sheaths infected with virulent *M. oryzae* RO1-1 to compare their effects on Ca^2+^ influx, ROS accumulation, lipid peroxidation, and HR cell death (**Figures 6**, **7B**; **Supplementary Figure 5**). ASM treatment significantly enhanced Ca^2+^ influx, ROS and iron accumulation and HR cell death in rice leaf sheath cells during virulent *M. oryzae* infection compared with the other tested Ca^2+^ influx enhancers (**Figures 6**, **7B**).

Other Ca^2+^ influx enhancers differentially induced Ca^2+^ influx, ROS accumulation, lipid peroxidation (MDA level) and HR cell death during virulent *M. oryzae* infection (**Figure 7B**; **Supplementary Figure 5**). Diamide, TFP, C/C, C/H, and BMA differentially induced Ca^2+^ influx; however, the induction of Ca^2+^ influx by rotenone was not significant. Diamide, TFP, C/C, C/H, and rotenone increased ROS levels by at least 15.98%. However, BMA did not affect ROS levels during infection. These Ca^2+^ influx enhancers also increased only slightly MDA levels in rice leaf sheath cells. However, diamide, TFP, C/C, C/H, rotenone, and BMA increased HR cell death up to 22.06-26.23% (**Figure 7B**). Prussian blue staining showed that Ca^2+^ influx enhancers effectively stimulated Fe^3+^ accumulation inside and around IH in rice cells during virulent *M. oryzae* infection (**Supplementary Figure 5**). These data indicate that Ca^2+^ influx enhancers trigger robust Ca^2+^ influx to varying degrees in compatible (susceptible) rice cells due to their different mode of actions, leading to iron- and ROS-dependent ferroptotic HR cell death in response to virulent *M. oryzae* infection (**Figures 6**, **7B**; **Supplementary Figure 5**).

### Ca^2+^ influx triggers reduced *OsGR* expression and GSH depletion in rice immune responses

Glutathione reductase (GR) reduces glutathione disulfide (GSSG; oxidized glutathione) to produce reduced glutathione (GSH) in the presence of NADPH which can be converted to NADP^+^ in plant cells (**Figure 8A**). *In vitro*, increasing Ca^2+^ concentration from 10 mM to 50 mM gradually inhibited the activity of rice GR or yeast GR (**Figure 8B**; **Supplementary Figures 6A, D**), indicating that higher Ca^2+^ concentration more effectively inhibits the reduction of GSSG to GSH by GR. Lineweaver-Burt plot showed non-competitive inhibition of GSSG reduction to GSH through rice GR or yeast GR by increasing Ca^2+^ concentration from 10 mM to 50 mM (**Supplementary Figures 6B, E**). Several metal ions, including Ca^2+^, Mg^2+^, K^+^, and Na^+^, which are abundant intracellular cations in plant cells, exhibited different inhibitory effects on the reduction of GSSG to GSH by rice GR or yeast GR *in vitro* (Tandoğan and Ulusu, 2007); however, Ca^2+^ was the most effective in inhibiting the reduction of GSSG to GSH (**Figure 8B**; **Supplementary Figures 6C, F**). This suggests that Ca^2+^ may more specifically inhibit *OsGR* activity and deplete GSH production than the other ions Mg^2+^, K^+^, and Na^+^ in rice cells.

The antioxidant enzyme glutathione reductase (GR) is responsible for maintaining the supply of reduced glutathione (GSH) for the cellular control of ROS inside cells (Couto et al., 2016). GR plays an important role in scavenging ROS to regulate the redox state of glutathione in plants (Wang et al., 2018). During avirulent *M. oryzae* infection, the expression of rice cytoplasmic *OsGR2* was effectively reduced relative to chloroplast *OsGR1* and *OsGR3* in leaf sheath cells compared with virulent *M. oryzae* infection (**Figure 8C**; **Supplementary Figure 7**). This indicates that avirulent *M. oryzae* infection significantly downregulates cytoplasmic *OsGR* expression to inhibit the reduction of GSSG to GSH. GSH depletion and lipid peroxidation are essential signaling events leading to iron- and lipid ROS-dependent ferroptotic cell death during avirulent *M. oryzae* infection (Dangol et al., 2019). During avirulent *M. oryzae* infection, treatment with the Ca^2+^ influx inhibitors EGTA and verapamil effectively rescued GSH depletion, increasing GSH content by 32.2%, and 28.3%, respectively (**Figure 8D**). The other Ca^2+^ influx inhibitors also more significantly increased GSH content compared with that of the control *M. oryzae* infection alone. However, during virulent *M. oryzae* infection, the Ca^2+^ influx enhancer ASM significantly decreased GSH production by 30.2% (**Figure 8D**). The other Ca^2+^ influx enhancers also more significantly reduced GSH production compared with that of the control *M. oryzae* infection alone.

## Discussion

Iron- and ROS-dependent ferroptotosis occurs not only in animals (Dixon et al., 2012), but also in plants (Dangol et al., 2019; Nguyen et al., 2022). Fe^2+^ present in the cell is highly reactive with ROS (H_2_O_2_), which subsequently produces Fe^3+^ and hydroxyl radicals (·OH) (Fenton, 1894; Pierre and Fontecave, 1999). Ferric ions (Fe^3+^) and ROS accumulated in rice tissues undergoing HR cell death during avirulent *M. oryzae* infection (Dangol et al., 2019). Iron- and ROS-dependent signaling is required for the ferroptotic cell death pathway in rice to disrupt *M. oryzae* infection. Rice iron storage protein ferritin 2 (OsFER2) could positively regulate rice ferroptosis and immune responses against *M. oryzae* infection (Nguyen et al., 2022). Mitogen-activated protein kinase (MAPK) signaling cascades are involved in plant immunity and HR cell death responses to pathogen infection (Meng and Zhang, 2013; Thulasi Devendrakumar et al., 2018). Rice MAP kinase (*OsMEK2* and *OsMPK1*) expression triggered iron- and ROS-dependent ferroptotic cell death (Dangol et al., 2021).

Calcium (Ca^2+^) is a secondary messenger that mediates diverse signaling pathways in eukaryotic cells (Zhao et al., 2021). In plants, Ca^2+^ influx is required for HR cell death in immune responses (Atkinson et al., 1990; Grant and Loake, 2000; Moeder et al., 2019). The ZAR1 resistosome is a calcium-permeable channel triggering plant immune signaling (Bi et al., 2021). Given the key role of calcium in plant immune responses and the emerging role of resistosomes as novel Ca^2+^ channels in plants, we hypothesized that Ca^2+^ acts as a key trigger of iron- and ROS-dependent ferroptotic cell death in rice immunity. In this study, we suggest that cytoplasmic Ca^2+^ influx through calcium-permeable cation channels, including the putative resistosomes, mediates iron- and lipid ROS-dependent ferroptotic cell death under reduced *OsGR* expression levels in rice immune responses.

### Reliable detection of Ca^2+^ influx in rice cells

To study the role of cytoplasmic Ca^2+^ influx in plant cells, it is absolutely necessary to accurately monitor fine spatial and temporal changes in intracellular Ca^2+^ concentration (Choi et al., 2012). The discovery and application of the Ca^2+^-sensitive bioluminescent photoprotein aequorin, made it possible to detect changes in cytoplasmic Ca^2+^ in the submicromolar range (Shimomura et al., 1963). However, despite advances in the Ca^2+^ detection techniques, the difficult detection and imaging of the weak luminescence signal of aequorin have been a limiting factor for its use in Ca^2+^ research (Hepler, 2005). Ca^2+^ indicators used for intracellular Ca^2+^ monitoring are mainly small molecules or proteins with highly specific sensitivity and responsiveness to intracellular Ca^2+^ (Kanchiswamy et al., 2014). The emerging role of resistosomes as Ca^2+^-permeable channels has stimulated the study of intracellular Ca^2+^ in plant immunity (Bi et al., 2021; Jacob et al., 2021). Accurate spatial and temporal Ca^2+^ imaging approaches are required to monitor resistsome-mediated Ca^2+^ influx into living rice cells during *M. oryzae* infection. Genetically encoded Ca^2+^ sensors that can be expressed inside the transgenic plant cells are preferably used because they do not require a staining step for Ca^2+^ detection.

In this study, we used fluorescence-based chemical Ca^2+^ indicator (Fluo-5F AM) and protein Ca^2+^ sensor (GCaMP6fmC), which are relatively more reliable compared to luminescence, to detect Ca^2+^ changes in living cells during cell death in rice immunity. The non-ratiometric Fluo Ca^2+^ indicator was first introduced to monitor Ca^2+^ influx in Lima bean leaf cells in response to herbivore attack (Maffei et al., 2004). Here, we selected Fluo-5F AM as a low-affinity intracellular Ca^2+^ indicator suitable for detecting high intracellular Ca^2+^ levels in the range of 1 μM to 1 mM in rice cells. We next used a genetically encoded ratiometric Ca^2+^ sensor (GCaMP6fmC) (Weigand et al., 2021) to investigate cytoplasmic Ca^2+^ influx in rice sheath cell during *M. oryzae* infection. The two different fluorescence-based Ca^2+^ detection tools were first shown to be highly reliable for detecting and quantifying changes in cytoplasmic Ca^2+^ levels in living rice cells during *M. oryzae* infection. In parallel with Ca^2+^ imaging, staining with Peroxy Orange 1 (PO1), a red fluorescent ROS indicator, revealed that the ROS burst occurs in the same region of the rice cell where Ca^2+^ influx occurs during avirulent *M. oryzae* infection. The red fluorescent ROS indicator PO1 could simultaneously visualize Ca^2+^ (green fluorescence) and ROS accumulation inside the same rice cell through its distinct red fluorescence emission.

### Ca^2+^ influx mediates ROS- and iron-dependent cell death in rice immunity

In the current study, we found that avirulent *M. oryzae* infection triggers a robust Ca^2+^ influx, which mediates iron- and ROS-dependent ferroptotic cell death in rice. The strong ROS burst is a cellular signaling event that occurs in HR cell death in plant immunity (Van Breusegem and Dat, 2006; Jwa and Hwang, 2017). Pathogen effectors can interact with NLR receptors of host plants, leading to ROS burst and HR cell death in plant immune responses (Jones and Dangl, 2006; Dangl et al., 2013; Han and Hwang, 2017). The ZAR1 resistosome has recently been proposed to be a calcium-permeable channel triggering plant immunity and cell death (Bi et al., 2021). Ca^2+^ influx from the apoplast to the cytoplasm may mediate iron- and ROS-dependent ferroptotic HR cell death in rice immunity. Plant cells maintain a steady state Ca^2+^ concentration of 100-200 nM in contrast to the Ca^2+^ concentration in the extracellular space at 1-10 mM (Martins et al., 2013). The steep Ca^2+^ concentration gradient between the cytoplasm (~100 nM) and apoplast (~1 mM) (Stael et al., 2012) may rapidly increase cytoplasmic Ca^2+^ influx through the resistosome, a membrane-localized Ca^2+^-permeable channel, which triggers calcium signaling of ROS- and iron-dependent ferroptotic cell death in rice immunity. Avirulent *M. oryzae* infection sustained high iron and ROS accumulation in ferroptotic HR cell death, with a layer of high Ca^2+^ concentration near the cell membrane, GSH depletion, and lipid peroxidation. The maintenance of a persistently high Ca^2+^ concentration gradient near the cell membrane is an ionic stress, which can have a significant impact on ambient ROS burst, iron accumulation, and antioxidant defense machineries. Ferroptotic HR cell death may only be caused by prolonged Ca^2+^/ROS bursts during ETI, but not transient Ca^2+^/ROS bursts during PTI (Grant and Loake, 2000). It is thus likely that continuous Ca^2+^ influx into the cytoplasm through the calcium-permeable cation channels, including the putative NLR resistosomes, mediates iron- and ROS-dependent ferroptotic HR cell death in the rice immune response.

### Ca^2+^ influx inhibitors block Ca^2+^-mediated iron- and ROS-dependent cell death

Different Ca^2+^ influx inhibitors such as EGTA, verapamil, NAC, neomycin, LiCl, AlCl3, and RR significantly suppressed cytoplasmic Ca^2+^ influx, ROS accumulation, glutathione (GSH) depletion, and lipid peroxidation, leading to reduced iron- and ROS-dependent ferroptotic HR cell death during avirulent *M. oryzae* infection. Notably, the membrane-impermeable Ca^2+^ chelator EGTA (Ellis-Davies and Kaplan, 1994; Cessna and Low, 2001) and the Ca^2+^ channel blocker verapamil (Beneloujaephajri et al., 2013) more effectively suppressed GSH depletion in rice sheaths than other Ca^2+^ influx inhibitors during avirulent *M. oryzae* infection. Ca^2+^ chelation by EGTA blocked Ca^2+^ influx from the apoplastic environment into the cytosol of rice cells during infection. EGTA can deplete extracellular free Ca^2+^ sources through high-affinity Ca^2+^ chelation, which essentially blocks Ca^2+^ influx from the apoplast. Resistosomes are thought to act as Ca^2+^-permeable channels that connect the cytoplasm to a high-level extracellular Ca^2+^ pool (Bi et al., 2021). The binding of Ca^2+^ to EGTA may prevent Ca^2+^ from freely diffusing across the cell membrane and consequently lower cytoplasmic Ca^2+^ levels.

Verapamil limits the transport of extracellular Ca^2+^ across the plasma membrane into the cytosol (Bergson et al., 2011). However, the inhibitory effect of verapamil on ferroptotic cell death was significantly lower than that of Ca^2+^ chelation with EGTA. NAC is a precursor of the antioxidant glutathione, which inhibits ROS levels and the oxidation of cellular glutathione (Sun et al., 2012). Neomycin acts as an inhibitor of polyphosphoinositide hydrolysis in animals and also inhibits Ca^2+^-dependent polyphosphoinositide-specific phospholipase C (PLC) activity in plants (Franklin-Tong et al., 1996). RR, a non-competitive inhibitor of the mitochondrial uniporter, is responsible for Ca^2+^ uptake into mitochondria in animal cells (Bae et al., 2003), and it also inhibits plant cell cation channels in a voltage-independent manner (White, 1996). Taken together, the seven different Ca^2+^ influx inhibitors tested in this study significantly inhibit Ca^2+^-mediated ROS, iron accumulation, GSH depletion, and lipid peroxidation, which ultimately increase the growth of invasive hyphae (IH) inside rice cells during avirulent *M. oryzae* infection leading to blast disease.

### Ca^2+^ influx enhancers promote Ca^2+^-mediated iron- and ROS-dependent cell death

Plant cells have high Ca^2+^ levels in both the apoplast and internal stores, and the cytoplasmic Ca^2+^ influx can increase intracellular Ca^2+^ concentration (Stael et al., 2012). Different Ca^2+^ influx enhancers ASM, diamide, TFP, CaCl_2_/calcimycin (C/C), CaCl_2_/H_2_O_2_ (C/H), rotenone, and BMA differentially induced Ca^2+^ influx, ROS accumulation, GSH depletion, lipid peroxidation (MDA level) leading to iron- and ROS-dependent ferroptotic HR cell death during virulent *M. oryzae* infection. The plant defense activator ASM, a salicylic acid (SA) analogue, induces systemic acquired resistance (SAR) against a broad spectrum of plant pathogens and is widely used for crop protection (Buonaurio et al., 2002; Matsuo et al., 2019). ASM significantly enhanced Ca^2+^ influx, as well as iron and ROS accumulation leading to ferroptotic cell death in rice even during virulent *M. oryzae* infection. ASM induced the highest increase in cytoplasmic Ca^2+^ level among the seven Ca^2+^ enhancers tested and effectively inhibited virulent *M. oryza*e infection. These results suggest that the enhancement of Ca^2+^ influx by ASM may activate SAR via resistosomes in plants, thereby contributing to disease control.

Diamide is a cell-permeable chemical oxidant that can specifically target thiols of GSH and free SH groups in proteins (Kosower et al., 1969; Gilge et al., 2008). Diamide treatment reduced intracellular GSH concentration as expected. The increase in Ca^2+^ levels induced a decrease in GSH, which increased ROS accumulation, lipid peroxidation, and HR cell death, resulting in a decrease in *M. oryzae* growth. These results suggest that reduced cytoplasmic GSH levels play a crucial role in iron- and lipid ROS-dependent HR cell death in rice immunity. TFP directly dissociates calmodulin (CaM) from IP_3_R [IP_3_ (inositol 1,4,5-trisphosphate) receptor] by interacting at the TFP-binding site of CaM and opens IP_3_R to release large amounts of Ca^2+^ from intracellular stores such as the endoplasmic reticulum (ER) in animals (Kang et al., 2017). Calcium ionophore A23187 (calcimycin), a hydrophobic molecule, can selectively bind to Ca^2+^ and permeate the hydrophobic interior of lipid bilayers, increasing cell permeability to Ca^2+^ (Verma et al., 2011). H_2_O_2_ is known to induce a biphasic cytoplasmic Ca^2+^ response in *Arabidopsis* seedling plants (Rentel and Knight, 2004). The recent discovery of a novel plant receptor that covalently regulates Ca^2+^ channel activity by H_2_O_2_ is a good example of direct activation of Ca^2+^ influx by H_2_O_2_ (Wu et al., 2020). Rotenone specifically targets mitochondrial complex 1 of the electron transport chain and causes impaired ATP production and oxidative stress, leading to cellular dysfunction and ultimately cell death (Li et al., 2003). BMA inhibits the dicarboxylate transporter (DIC), which transports glutathione into mitochondria in animal cells (Kamga et al., 2010). Taken together, Ca^2+^ influx enhancers could effectively promote cytoplasmic Ca^2+^ influx to trigger iron- and ROS-dependent ferroptotic cell death in rice plants during virulent *M. oryzae* infection.

### Ca^2+^ influx reduced GR expression that causes GSH depletion, and iron- and lipid ROS-dependent cell death

Ca^2+^ may operate more like an essential switch to signal plant cell death and immunity (Scrase-Field and Knight, 2003). Glutathione reductase (GR) catalyzes the NADPH-dependent reduction of oxidized glutathione (GSSG) to reduced glutathione (GSH) (Tandoğan and Ulusu, 2007). Robust Ca^2+^ influx into the cytoplasm triggered reduced *OsGR* expression and GSH depletion during avirulent *M. oryzae* infection. Inhibition of cytoplasmic *OsGR2* expression by avirulent *M. oryzae* infection may cause GSH depletion, leading to rice ferroptotic cell death. The intracellular concentration of glutathione can indicate the condition of oxidative stress in cells (Pastore et al., 2001). Within the cell, glutathione exists in a reduced state (GSH) and an oxidized state (GSSG). Glutathione, an important cellular antioxidant, plays a crucial role in disease resistance in plants by helping to regulate intracellular ROS homeostasis (Hwang et al., 1992; Parisy et al., 2007). GSH depletion disrupts intracellular ROS homeostasis and leads to iron- and ROS-dependent ferroptotic cell death (Dangol et al., 2019). GSH depletion is a common phenomenon observed during both plant and animal ferroptosis (Stockwell et al., 2017; Dangol et al., 2019).

An increase in free Ca^2+^ levels in apoplasts during avirulent *M. oryzae* infection may cause Ca^2+^ influx into the cytoplasm via calcium-permeable cation channels, including the putative NLR-activated resistosomes. High cytoplasmic Ca^2+^ influx can inhibit the reduction of oxidized glutathione (GSSG) to GSH by GR, leading to GSH depletion *in vitro*. Cytoplasmic Ca^2+^ influx from the apoplast through the putative resistosomes induces GSH depletion, which leads to the production of lipid ROS, thus promoting iron-dependent ferroptotic cell death in rice. Robust Ca^2+^, ROS and iron accumulation occurred together in the vicinity of the plasma membrane, where resistosomes are likely localized, during avirulent *M. oryzae* infection. This highlights the importance of GSH depletion for the initiation of Ca^2+^-mediated ferroptotic cell death in rice during *M. oryzae* infection. Strong Ca^2+^ influx into the cytoplasm leading to GSH depletion may be due primarily to the irreversible influx of apoplastic Ca^2+^ through the putative resistosomes, Ca^2+^-permeable selective channels (Bi et al., 2021), in the plasma membrane. It is thus likely that the irreversible Ca^2+^ influx acts as a driving force for lipid ROS production that induces ferroptotic cell death.

**Figure 9:**
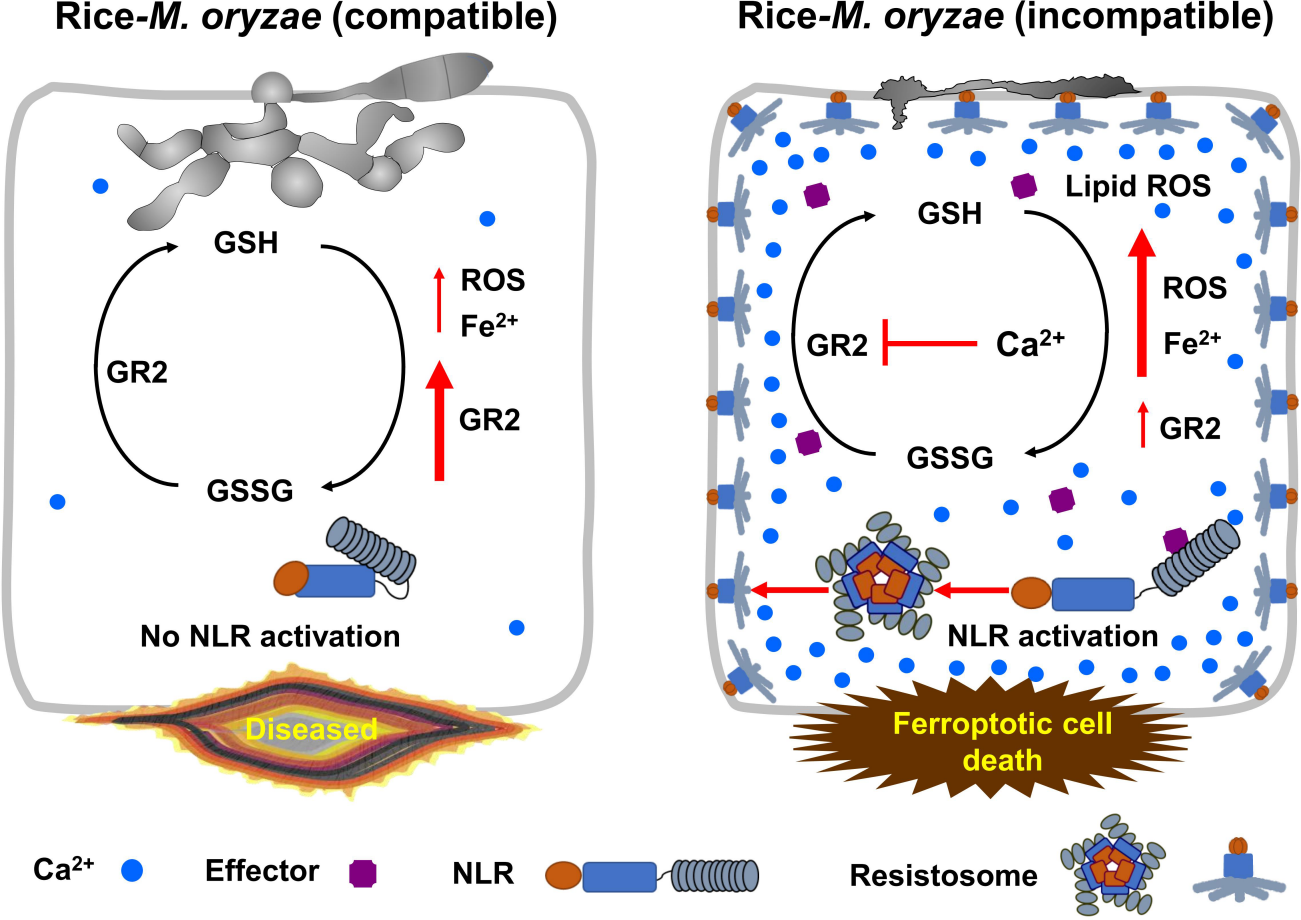
Model of Ca^2+^-mediated iron- and lipid ROS-dependent ferroptotic cell death by the putative NLR-activated resistosomes in rice immune responses. Glutathione is an important and potent antioxidant that regulates intracellular ROS homeostasis through the continuous redox. In compatible rice-*M. oryzae* (virulent) interactions, massive cytoplasmic Ca^2+^-influx does not occur due to inactivation of NLR, ROS homeostasis is well maintained by redox of glutathione due to increased expression of *glutathione reductase* (*GR*), and *M. oryzae* hyphae grow normally and successfully infect rice. By contrast, during incompatible rice-*M. oryzae* (avirulent) interactions, plant NLR-pathogen effector recognition leads to resistosome formation, and resistosome-mediated massive influx of cytoplasmic Ca^2+^ from the apoplast sharply represses *GR* expression in rice cells during the early infection stage. Consequently, reduced GR activity exacerbates glutathione depletion, leading to iron- and lipid ROS-dependent ferroptotic cell death in the rice immune response. GR, glutathione reductase; GSSG, glutathione disulfide; GSH, reduced glutathione; NLR, nucleotide-binding leucine-rich repeat receptor.

Our data collectively support a model of Ca^2+^-mediated iron- and lipid ROS-dependent ferroptotic cell death by calcium-permeable cation channels, including the putative NLR-activated resistosomes and consequent GSH depletion during *M. oryzae* infection in rice (**Figure 9**). Recently, multiple major NLR genes, including *PigmR*, which confer broad-spectrum resistance to *M. oryzae* have been identified in the rice genome (Deng et al., 2017; Wang L. et al., 2019; Zhai et al., 2019). Plant NLRs form resistosomes upon the recognition of pathogen effectors (Bi et al., 2021); however, there is still no experimental evidence that rice NLRs such as *PigmR* form resistosomes that recognize *M. oryzae* effectors. The NLR resistosomes (Wang J. et al., 2019) act as irreversible Ca^2+^-permeable channels in the plasma membrane to trigger HR cell death in plant immunity (Bi et al., 2021). Cytoplasmic Ca^2+^ influx by calcium-permeable cation channels, including the putative resistosome induces GSH depletion and eventually triggers a ROS burst at the same site where Ca^2+^ accumulation occurs. During virulent *M. oryzae* infection, NLR-activated resistosome formation and GSH depletion do not occur due to increased expression of rice *glutathione reductase* (*OsGR*), and *M. oryzae* hyphae grow invasively inside rice cells, causing disease without ferroptotic cell death. Blast disease (susceptibility)-related cell death is ROS-dependent but iron-independent in the compatible rice–*M. oryzae* interaction (Dangol et al., 2021). However, during avirulent *M. oryzae* infection, strong cytoplasmic Ca^2+^ influx through calcium-permeable cation channels, including the putative NLR-activated resistosomes triggers iron- and ROS-dependent ferroptotic cell death owing to the increase in Fe^2+^, ROS and lipid ROS levels as well as GSH depletion by repressed *OsGR2* expression. Overall, our results suggest that cytoplasmic Ca^2+^ influx from the apoplast through calcium-permeable cation channels, including the putative NLR resistosomes (Wang J. et al., 2019) inhibits the reduction of GSSG to GSH under reduced *OsGR* expression levels, leading to iron- and lipid ROS-dependent ferroptotic cell death in the rice immune response.

## Data availability statement

The original contributions presented in the study are included in the article/**Supplementary Materials**, further inquiries can be directed to the corresponding author/s.

## Author contributions

JW: Conceptualization, Data curation, Formal analysis, Investigation, Methodology, Visualization, Writing – original draft, Writing – review & editing. W-GC: Formal analysis, Funding acquisition, Investigation, Methodology, Software, Validation, Visualization, Writing – review & editing. NKN: Data curation, Formal analysis, Investigation, Methodology, Validation, Visualization, Writing – review & editing. DPL: Data curation, Formal analysis, Investigation, Methodology, Validation, Visualization, Writing – review & editing. S-HK: Formal analysis, Investigation, Methodology, Validation, Writing – review & editing. DYL: Formal analysis, Methodology, Validation, Writing – review & editing. BKH: Conceptualization, Formal analysis, Methodology, Supervision, Validation, Visualization, Writing – original draft, Writing – review & editing. N-SJ: Conceptualization, Data curation, Formal analysis, Funding acquisition, Investigation, Methodology, Project administration, Resources, Software, Supervision, Validation, Visualization, Writing – original draft, Writing – review & editing.

## Supplementary material

Supplementary Figure 1 Analysis of the effect of Ca^2+^ influx on the immunity of avirulent *Magnaprthe oryzae* 007-inoculated rice using the transgenic approach. **(A)** Schematic diagram of the Ca^2+^ sensor construct. The Ca^2+^ sensor GCaMP6fmC is composed of the calmodulin (CaM)-binding site M13, a circularly permutated enhanced green fluorescent protein (cp-eGFP), and mCherry-fused CaM. The Ca^2+^ sensor construct *GCaMP6f-mCherry* (*GCaMP6fmC*) was cloned into the vector pGWB554 under the control of the Cauliflower mosaic virus (CaMV) *35S* promoter. LB, left border; RB, right border. **(B)** PCR confirmation of Ca^2+^ sensor (GCaMP6fmC) transgenic lines of the rice cultivar Kitaake using hygromycin resistance (HygR) and GCaMP6fmC primers. **(C)** Detection of Ca^2+^ influx in rice leaf sheaths during avirulent *M. oryzae* 007 infection. The leaf sheaths of wild-type (WT) plants and Ca^2+^ sensor (GCaMP6fmC) transgenic lines A, B, and C were inoculated with avirulent *M. oryzae* 007, and Ca^2+^ influx in leaf sheath cells was visualized by fluorescence microscopy at 36 h post-inoculation (hpi). Images were taken using a microscope (Zeiss equipped with Axioplan 2) with bright field filter, green fluorescence filter (Ex/Em: 450–490/515–565 nm), and red fluorescence filter (Ex/Em: 546/590 nm). Bars = 10 µm. BF, bright field; GF, green fluorescence; RF, red fluorescence.

Supplementary Figure 2 Images of Ca^2+^ influx and ROS and iron accumulation in the leaf sheaths of rice Kitaake (WT) and *35S::GCamP6fmC* line B plants during *Magnaporthe oryzae* infection. The leaf sheaths of WT and Ca^2+^ sensor (GCaMP6fmC) transgenic line B plants were inoculated with *M. oryzae* RO1-1 (virulent) and 007 (avirulent), and the influx of Ca^2+^, accumulation of ROS (H_2_O_2_), and accumulation of ferric ions (Fe^3+^) were measured at 36, 40, and 48 hpi, respectively. Rice leaf sheath cells were stained with Peroxy Orange 1 (PO1) to visualize H_2_O_2_ accumulation around the site of infection. Fe^3+^ accumulation was detected by Prussian blue staining. Ca^2+^ influx, H_2_O_2_ accumulation, and Fe^3+^ accumulation in rice leaf sheaths were observed under a microscope (Zeiss equipped with Axioplan 2) using a bright field filter and/or fluorescence filters. Bars = 10 µm. Red arrows indicate invasive hyphae. WT, wild type; BF, bright field; GF, green fluorescence; RF, red fluorescence. hpi, hours post-inoculation.

Supplementary Figure 3 Ca^2+^ chelation by EGTA suppresses apoplastic Ca^2+^ influx in rice leaf sheaths during avirulent *Magnaporthe oryzae* 007 infection. **(A)** Schematic diagram of apoplastic Ca^2+^ detection using the calcium-*o*-cresolphthalein complexone (*o*-CPC) method. The binding of Ca^2+^ to *o*-CPC results in the formation of an intense violet-colored complex, which can be quantified by measuring the absorbance of the intercellular fluid of the rice leaf sheath at 575 nm using the SP-2000UV spectrophotometer. **(B)** Quantitative determination of apoplastic Ca^2+^ concentrations in rice leaf sheaths treated with 10 mM EGTA during avirulent *M. oryzae* 007 infection. The leaf sheaths of rice (Kitaake) plants were inoculated with avirulent *M. oryzae* 007 (4 × 10^5^ conidia·mL^-1^) supplemented with 10 mM EGTA. Ca^2+^ in the intercellular fluid of rice leaf sheath was detected using the *o*-CPC method, and Ca^2+^ concentration was measured at 575 nm using the SP-2000UV spectrophotometer. Data are represented as the mean ± SD (*n* = 4 leaf sheaths from different plants). Asterisks above bars indicate significantly different means (**P*< 0.05, ***P*< 0.01; Student’s *t*-test). Experiments were repeated three times with similar results.

Supplementary Figure 4 Different Ca^2+^ influx inhibitors significantly limit Ca^2+^-mediated iron- and ROS-dependent ferroptotic cell death in rice immune responses. Images show HR cell death (48 hpi), Ca^2+^ influx (36 hpi), ROS accumulation (36 hpi), and Fe^3+^ accumulation (48 hpi) in the leaf sheath cells of rice (Kitaake) Ca^2+^ sensor (GCaMP6fmC) transgenic line B infected with *M. oryzae* 007 (avirulent), supplemented with Ca^2+^ influx inhibitors, including verapamil hydrochloride (verapamil), N-acetyl-cysteine (NAC), neomycin sulfate (neomycin), lithium chloride (LiCl), aluminum chloride (AlCl_3_), and ruthenium red (RR). HR, hypersensitive response; BF, bright field; GF, green fluorescence; RF, red fluorescence; hpi, hours post-inoculation. Bars = 10 µm. Different Ca^2+^ influx inhibitors significantly limit Ca^2+^-mediated iron- and ROS-dependent ferroptotic cell death in rice immune responses. Images show HR cell death (48 hpi), Ca^2+^ influx (36 hpi), ROS accumulation (36 hpi), and Fe^3+^ accumulation (48 hpi) in the leaf sheath cells of rice (Kitaake) Ca^2+^ sensor (GCaMP6fmC) transgenic line B infected with *M. oryzae* 007 (avirulent), supplemented with Ca^2+^ influx inhibitors, including verapamil hydrochloride (verapamil), N-acetyl-cysteine (NAC), neomycin sulfate (neomycin), lithium chloride (LiCl), aluminum chloride (AlCl_3_), and ruthenium red (RR). HR, hypersensitive response; BF, bright field; GF, green fluorescence; RF, red fluorescence; hpi, hours post-inoculation. Bars = 10 µm.

Supplementary Figure 5 Different Ca^2+^ influx enhancers significantly trigger Ca^2+^-mediated iron- and ROS-dependent ferroptotic cell death in rice immune responses. Images show HR cell death (48 hpi), Ca^2+^ influx (36 hpi), ROS accumulation (36 hpi), and Fe^3+^ accumulation (48 hpi) in the leaf sheath cells of rice (Kitaake) Ca^2+^ sensor (GCaMP6fmC) transgenic line B infected with *M. oryzae* RO1-1 (virulent), supplemented with Ca^2+^ influx enhancers, including diamide, trifluoperazine dihydrochloride (TFP), CaCl_2_/calcimycin (C/C), CaCl_2_/H_2_O_2_ (C/H), rotenone and butylmalonic acid (BMA). HR, hypersensitive response; BF, bright field; GF, green fluorescence; RF, red fluorescence; hpi, hours post inoculation. Bars = 10 µm.

Supplementary Figure 6 High concentrations of CaCl_2_ inhibit the reduction of GSSG to GSH. GSSG can be reduced to GSH by glutathione reductase (GR) using NADPH as a cofactor, which is converted to NADP^+^ during the reaction. The decrease in the absorbance of NADPH at 340 nm was measured spectrophotometrically for 5 min at room temperature. **(A, D)** Effect of the increase in CaCl_2_ concentration on the inhibition of GSSG reduction to GSH by rice GR **(A)** or yeast GR **(D)**. Values represent mean ± SD of OD_340_ at different time points. **(B, E)** Linerweaver-Burt plot showing non-competitive inhibition of the GSSG reduction reaction by rice GR **(B)** or yeast GR **(E)**. The x-axis represents the inverse of the substrate concentration (1/S), and the y-axis represents the inverse of the initial reaction velocity (1/V_0_). **(C, F)** Inhibitory effects of CaCl_2_, MgCl_2_, KCl, and NaCl on GSSG reduction to GSH by rice GR (**C)** or yeast GR **(F)**. Values represent mean ± SD of OD_340_ values at different time points.

Supplementary Figure 7 RT-PCR and q-RT-PCR analyses of the expression levels of rice glutathione reductase (*OsGR*) genes in the leaf sheaths of rice (Kitaake) plants during *Magnaporthe oryzae* infection. **(A)** Reverse transcription PCR (RT-PCR) analysis of the expression levels of *OsGR1*, *OsGR2*, *OsGR3*, and *Ubiquitin* (*OsUbiquitin*) in rice leaf sheaths at different time points after inoculation with *M. oryzae* RO1-1 (virulent) and 007 (avirulent). **(B)** Real-time quantitative RT-PCR (qRT-PCR) analysis of the expression levels of *OsGR1*, *OsGR3*, and *OsUbiquitin* in rice leaf sheaths at different time points after inoculation with *M. oryzae* RO1-1 (virulent) and 007 (avirulent). Transcription levels of *OsGR* genes were normalized relative to that of the internal reference gene *OsUbiquitin*. hpi, hours post-inoculation.
