# Supplementary figures and images for: Cytoplasmic Ca2+ influx mediates iron- and reactive oxygen species-dependent ferroptotic cell death in rice immunity
Source: Front Plant Sci. 2024 May 2;15:1339559. doi: 10.3389/fpls.2024.1339559 (PMC11096502; doi:10.3389/fpls.2024.1339559)

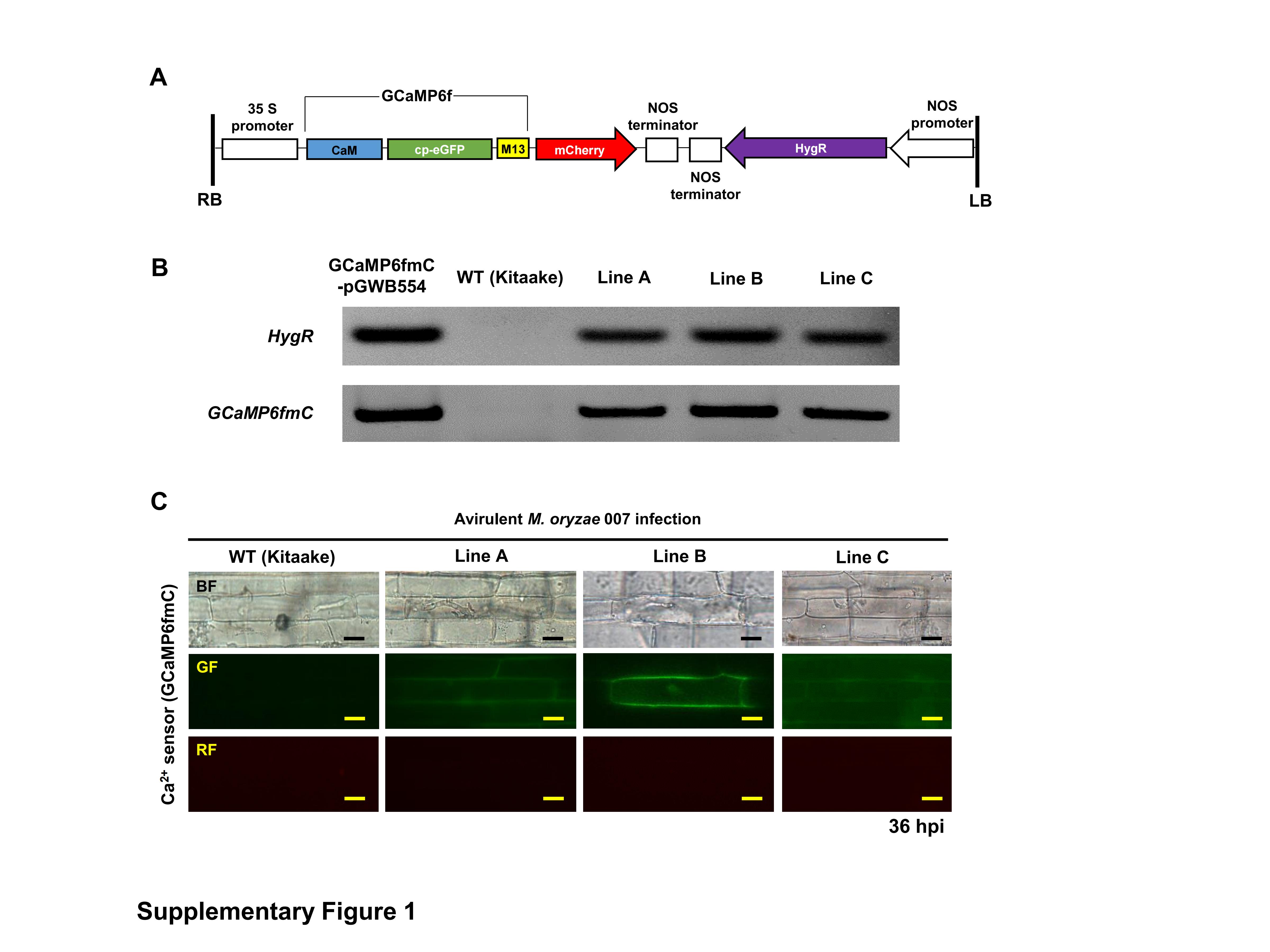

Supplement: Supplementary Figure 1 — Analysis of the effect of Ca2+ influx on the immunity of avirulent Magnaprthe oryzae 007-inoculated rice using the transgenic approach. (A) Schematic diagram of the Ca2+ sensor construct. The Ca2+ sensor GCaMP6fmC is composed of the calmodulin (CaM)-binding site M13, a circularly permutated enhanced green fluorescent protein (cp-eGFP), and mCherry-fused CaM. The Ca2+ sensor construct GCaMP6f-mCherry (GCaMP6fmC) was cloned into the vector pGWB554 under the control of the Cauliflower mosaic virus (CaMV) 35S promoter. LB, left border; RB, right border. (B) PCR confirmation of Ca2+ sensor (GCaMP6fmC) transgenic lines of the rice cultivar Kitaake using hygromycin resistance (HygR) and GCaMP6fmC primers. (C) Detection of Ca2+ influx in rice leaf sheaths during avirulent M. oryzae 007 infection. The leaf sheaths of wild-type (WT) plants and Ca2+ sensor (GCaMP6fmC) transgenic lines A, B, and C were inoculated with avirulent M. oryzae 007, and Ca2+ influx in leaf sheath cells was visualized by fluorescence microscopy at 36 h post-inoculation (hpi). Images were taken using a microscope (Zeiss equipped with Axioplan 2) with bright field filter, green fluorescence filter (Ex/Em: 450–490/515–565 nm), and red fluorescence filter (Ex/Em: 546/590 nm). Bars = 10 µm. BF, bright field; GF, green fluorescence; RF, red fluorescence. [file Image_1.jpeg]

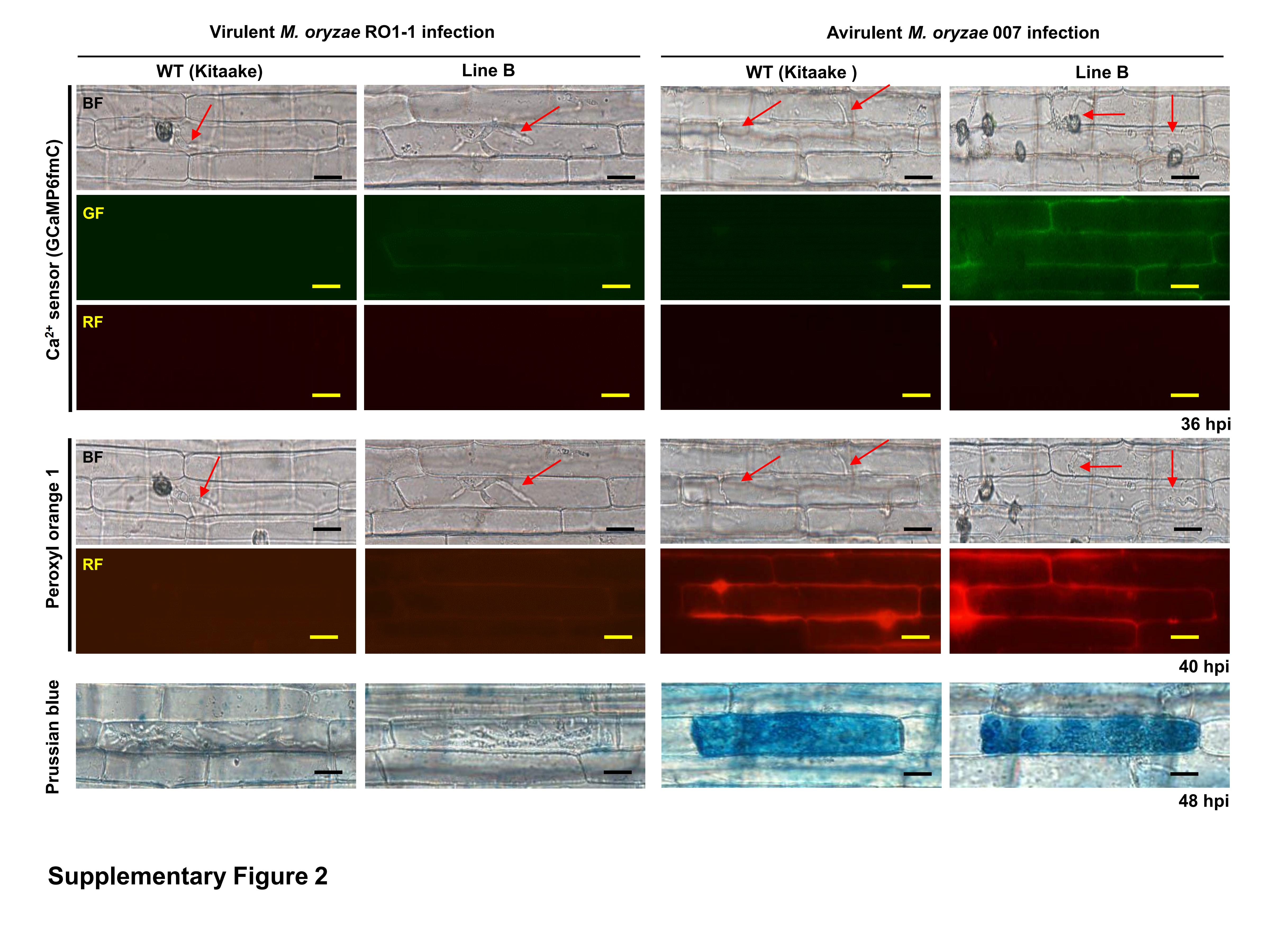

Supplement: Supplementary Figure 2 — Images of Ca2+ influx and ROS and iron accumulation in the leaf sheaths of rice Kitaake (WT) and 35S::GCamP6fmC line B plants during Magnaporthe oryzae infection. The leaf sheaths of WT and Ca2+ sensor (GCaMP6fmC) transgenic line B plants were inoculated with M. oryzae RO1-1 (virulent) and 007 (avirulent), and the influx of Ca2+, accumulation of ROS (H2O2), and accumulation of ferric ions (Fe3+) were measured at 36, 40, and 48 hpi, respectively. Rice leaf sheath cells were stained with Peroxy Orange 1 (PO1) to visualize H2O2 accumulation around the site of infection. Fe3+ accumulation was detected by Prussian blue staining. Ca2+ influx, H2O2 accumulation, and Fe3+ accumulation in rice leaf sheaths were observed under a microscope (Zeiss equipped with Axioplan 2) using a bright field filter and/or fluorescence filters. Bars = 10 µm. Red arrows indicate invasive hyphae. WT, wild type; BF, bright field; GF, green fluorescence; RF, red fluorescence. hpi, hours post-inoculation. [file Image_2.jpeg]

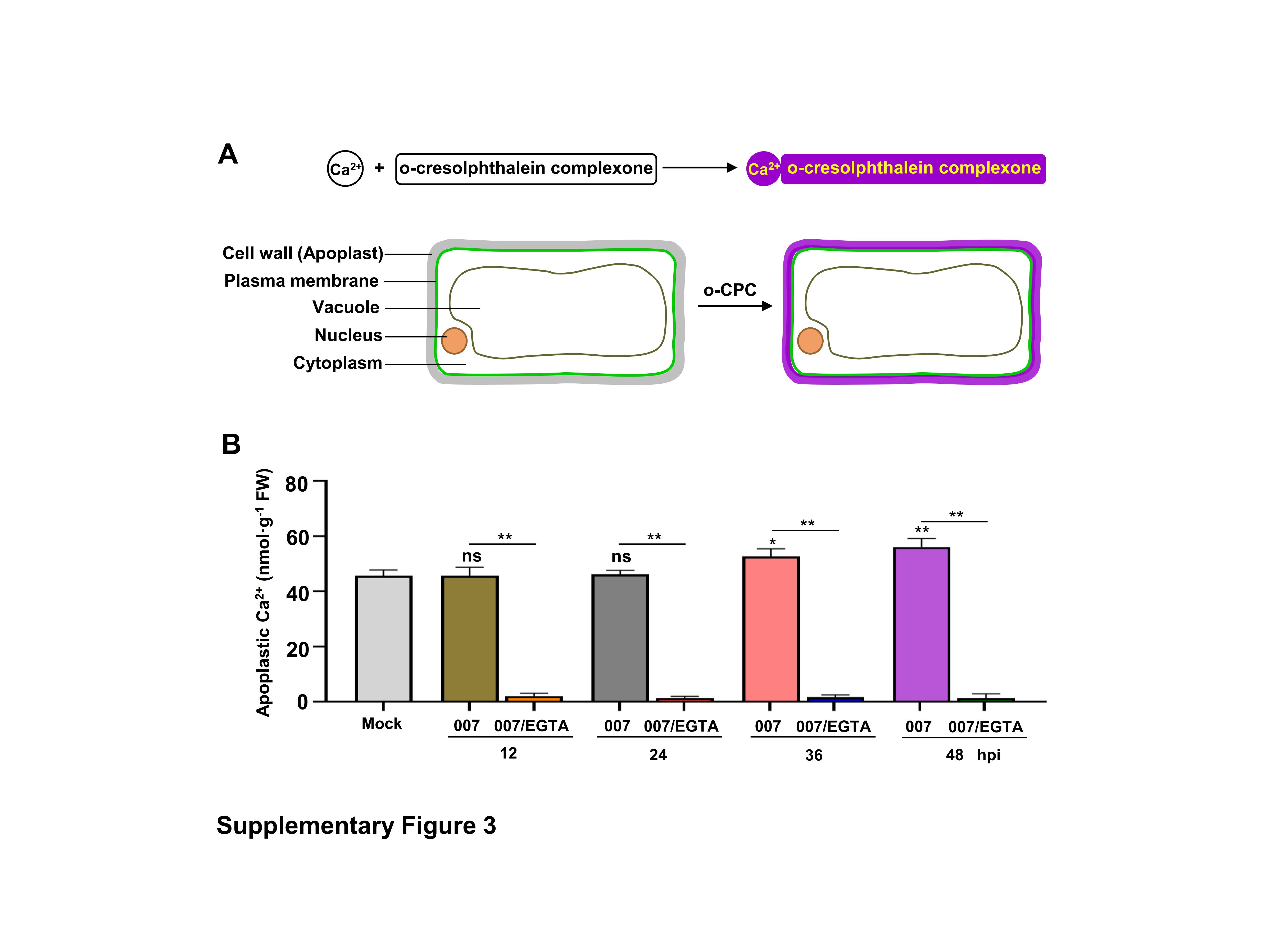

Supplement: Supplementary Figure 3 — Ca2+ chelation by EGTA suppresses apoplastic Ca2+ influx in rice leaf sheaths during avirulent Magnaporthe oryzae 007 infection. (A) Schematic diagram of apoplastic Ca2+ detection using the calcium-o-cresolphthalein complexone (o-CPC) method. The binding of Ca2+ to o-CPC results in the formation of an intense violet-colored complex, which can be quantified by measuring the absorbance of the intercellular fluid of the rice leaf sheath at 575 nm using the SP-2000UV spectrophotometer. (B) Quantitative determination of apoplastic Ca2+ concentrations in rice leaf sheaths treated with 10 mM EGTA during avirulent M. oryzae 007 infection. The leaf sheaths of rice (Kitaake) plants were inoculated with avirulent M. oryzae 007 (4 × 10^5^ conidia·mL-1) supplemented with 10 mM EGTA. Ca2+ in the intercellular fluid of rice leaf sheath was detected using the o-CPC method, and Ca2+ concentration was measured at 575 nm using the SP-2000UV spectrophotometer. Data are represented as the mean ± SD (n = 4 leaf sheaths from different plants). Asterisks above bars indicate significantly different means (*P< 0.05, **P< 0.01; Student’s t-test). Experiments were repeated three times with similar results. [file Image_3.jpeg]

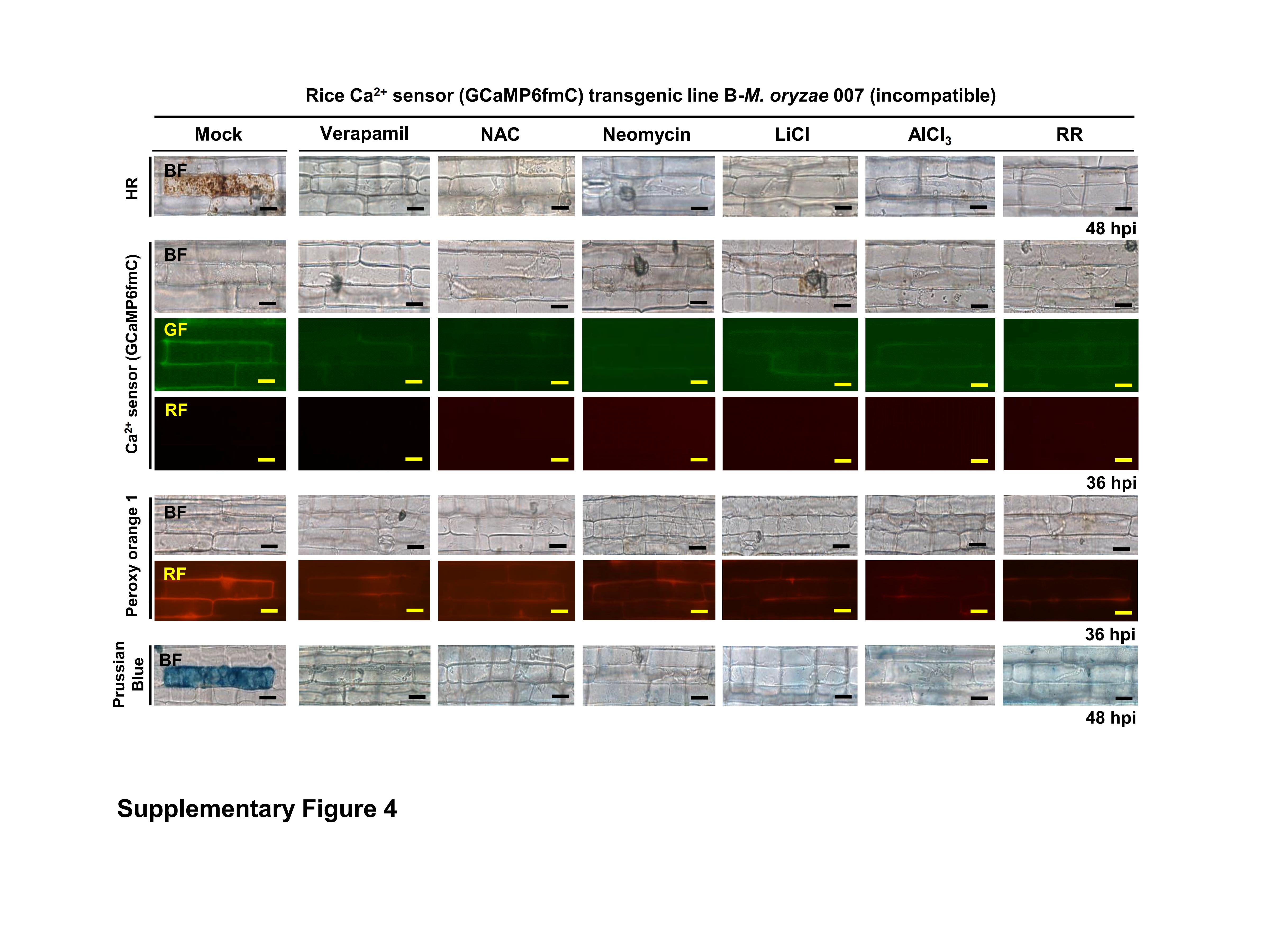

Supplement: Supplementary Figure 4 — Different Ca2+ influx inhibitors significantly limit Ca2+-mediated iron- and ROS-dependent ferroptotic cell death in rice immune responses. Images show HR cell death (48 hpi), Ca2+ influx (36 hpi), ROS accumulation (36 hpi), and Fe3+ accumulation (48 hpi) in the leaf sheath cells of rice (Kitaake) Ca2+ sensor (GCaMP6fmC) transgenic line B infected with M. oryzae 007 (avirulent), supplemented with Ca2+ influx inhibitors, including verapamil hydrochloride (verapamil), N-acetyl-cysteine (NAC), neomycin sulfate (neomycin), lithium chloride (LiCl), aluminum chloride (AlCl3), and ruthenium red (RR). HR, hypersensitive response; BF, bright field; GF, green fluorescence; RF, red fluorescence; hpi, hours post-inoculation. Bars = 10 µm. Different Ca2+ influx inhibitors significantly limit Ca2+-mediated iron- and ROS-dependent ferroptotic cell death in rice immune responses. Images show HR cell death (48 hpi), Ca2+ influx (36 hpi), ROS accumulation (36 hpi), and Fe3+ accumulation (48 hpi) in the leaf sheath cells of rice (Kitaake) Ca2+ sensor (GCaMP6fmC) transgenic line B infected with M. oryzae 007 (avirulent), supplemented with Ca2+ influx inhibitors, including verapamil hydrochloride (verapamil), N-acetyl-cysteine (NAC), neomycin sulfate (neomycin), lithium chloride (LiCl), aluminum chloride (AlCl3), and ruthenium red (RR). HR, hypersensitive response; BF, bright field; GF, green fluorescence; RF, red fluorescence; hpi, hours post-inoculation. Bars = 10 µm. [file Image_4.jpeg]

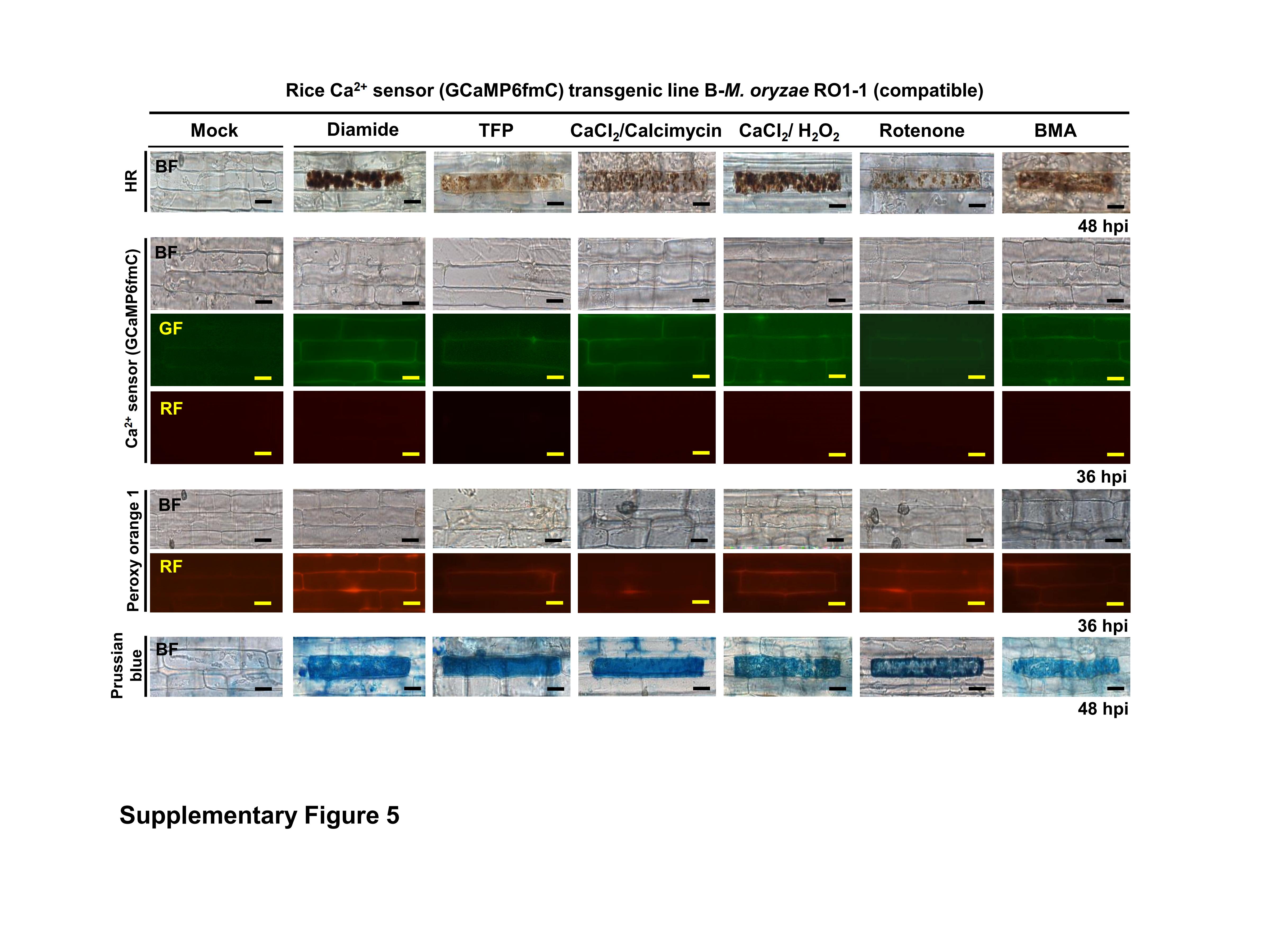

Supplement: Supplementary Figure 5 — Different Ca2+ influx enhancers significantly trigger Ca2+-mediated iron- and ROS-dependent ferroptotic cell death in rice immune responses. Images show HR cell death (48 hpi), Ca2+ influx (36 hpi), ROS accumulation (36 hpi), and Fe3+ accumulation (48 hpi) in the leaf sheath cells of rice (Kitaake) Ca2+ sensor (GCaMP6fmC) transgenic line B infected with M. oryzae RO1-1 (virulent), supplemented with Ca2+ influx enhancers, including diamide, trifluoperazine dihydrochloride (TFP), CaCl2/calcimycin (C/C), CaCl2/H2O2 (C/H), rotenone and butylmalonic acid (BMA). HR, hypersensitive response; BF, bright field; GF, green fluorescence; RF, red fluorescence; hpi, hours post inoculation. Bars = 10 µm. [file Image_5.jpeg]

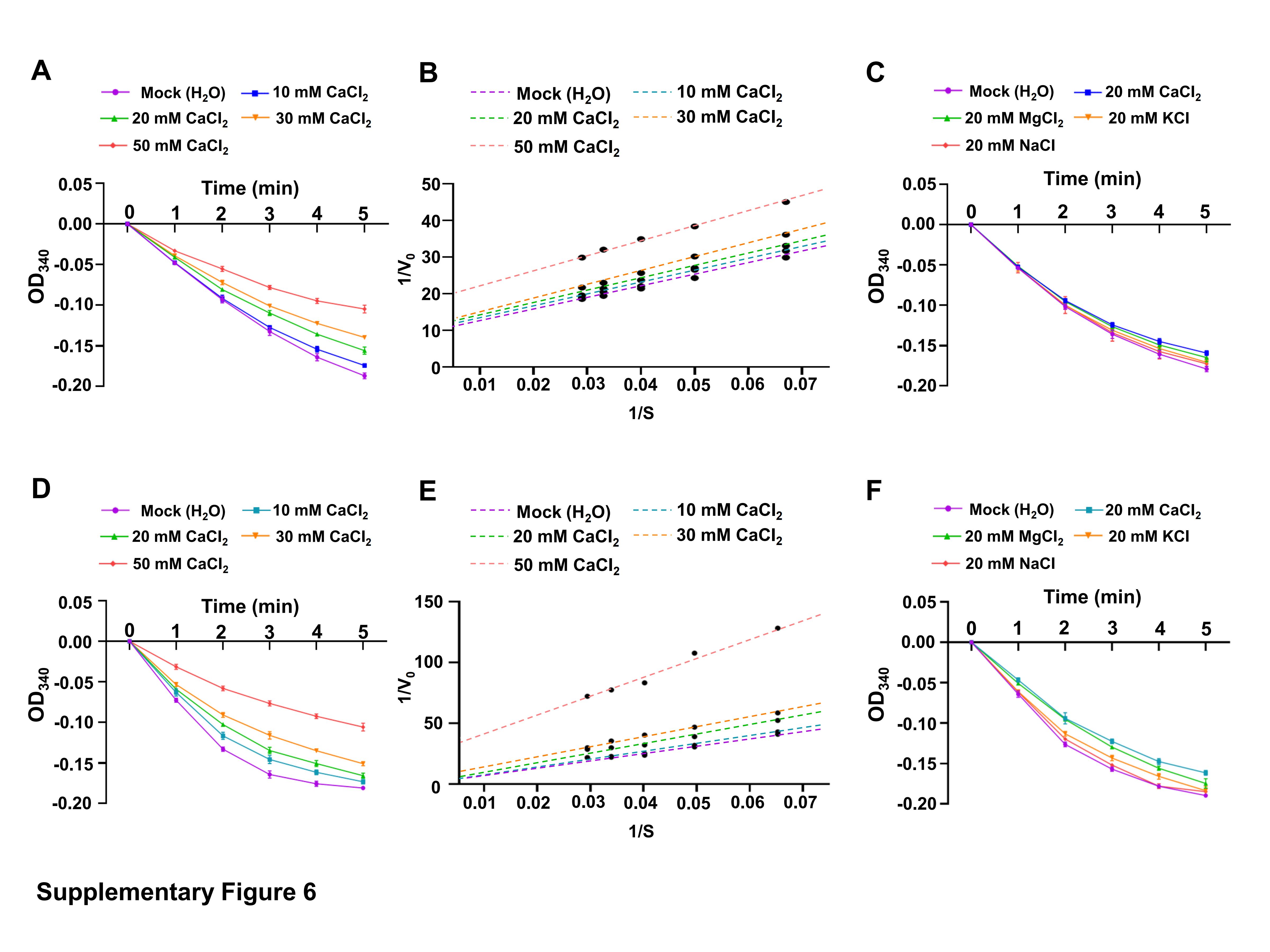

Supplement: Supplementary Figure 6 — High concentrations of CaCl2 inhibit the reduction of GSSG to GSH. GSSG can be reduced to GSH by glutathione reductase (GR) using NADPH as a cofactor, which is converted to NADP+ during the reaction. The decrease in the absorbance of NADPH at 340 nm was measured spectrophotometrically for 5 min at room temperature. (A, D) Effect of the increase in CaCl2 concentration on the inhibition of GSSG reduction to GSH by rice GR (A) or yeast GR (D). Values represent mean ± SD of OD340 at different time points. (B, E) Linerweaver-Burt plot showing non-competitive inhibition of the GSSG reduction reaction by rice GR (B) or yeast GR (E). The x-axis represents the inverse of the substrate concentration (1/S), and the y-axis represents the inverse of the initial reaction velocity (1/V0). (C, F) Inhibitory effects of CaCl2, MgCl2, KCl, and NaCl on GSSG reduction to GSH by rice GR (C) or yeast GR (F). Values represent mean ± SD of OD340 values at different time points. [file Image_6.jpeg]

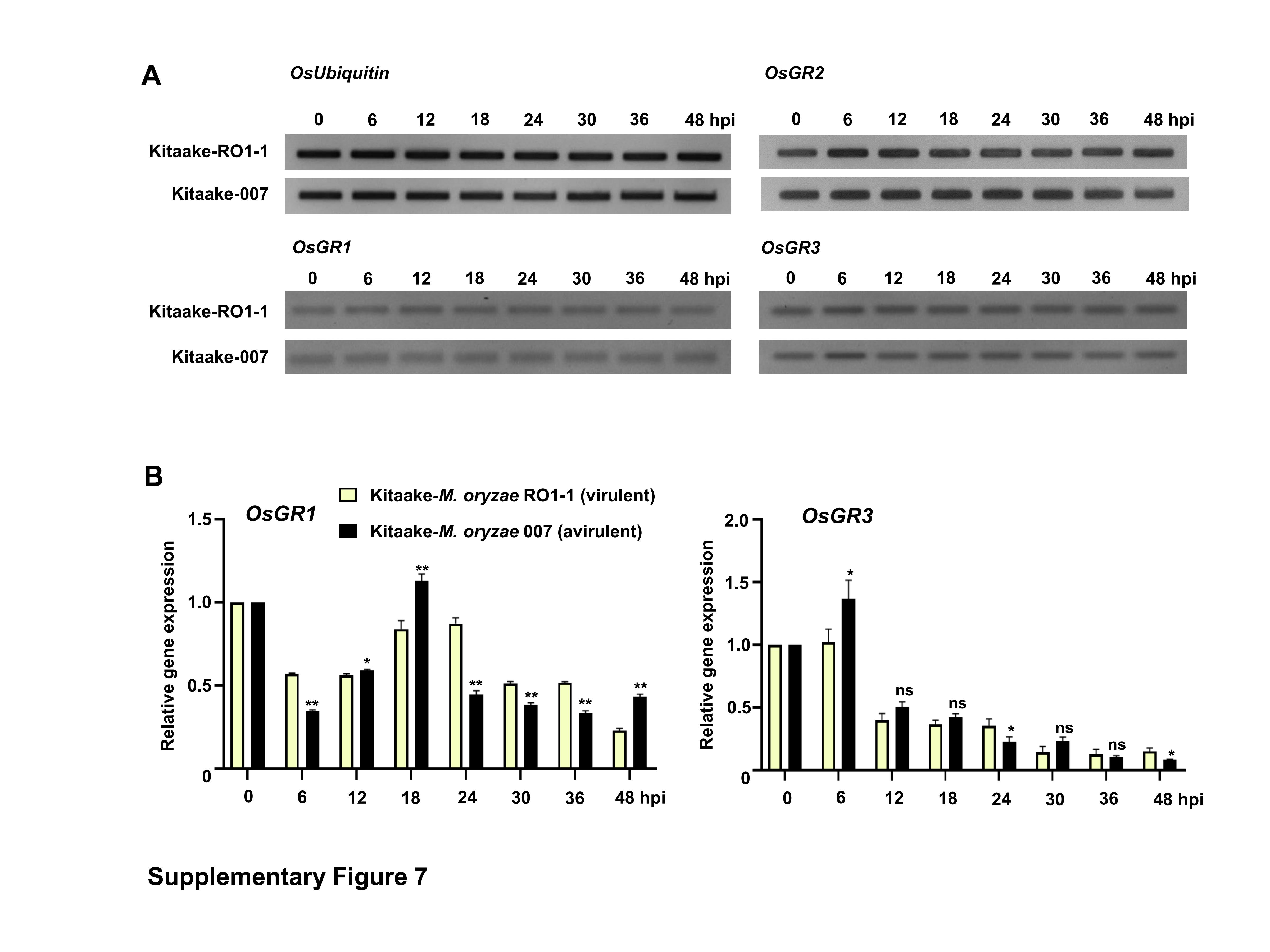

Supplement: Supplementary Figure 7 — RT-PCR and q-RT-PCR analyses of the expression levels of rice glutathione reductase (OsGR) genes in the leaf sheaths of rice (Kitaake) plants during Magnaporthe oryzae infection. (A) Reverse transcription PCR (RT-PCR) analysis of the expression levels of OsGR1, OsGR2, OsGR3, and Ubiquitin (OsUbiquitin) in rice leaf sheaths at different time points after inoculation with M. oryzae RO1-1 (virulent) and 007 (avirulent). (B) Real-time quantitative RT-PCR (qRT-PCR) analysis of the expression levels of OsGR1, OsGR3, and OsUbiquitin in rice leaf sheaths at different time points after inoculation with M. oryzae RO1-1 (virulent) and 007 (avirulent). Transcription levels of OsGR genes were normalized relative to that of the internal reference gene OsUbiquitin. hpi, hours post-inoculation. [file Image_7.jpeg]
